# Positive selection alone is sufficient for whole genome differentiation at the early stage of speciation process in the fall armyworm

**DOI:** 10.1186/s12862-020-01715-3

**Published:** 2020-11-13

**Authors:** Kiwoong Nam, Sandra Nhim, Stéphanie Robin, Anthony Bretaudeau, Nicolas Nègre, Emmanuelle d’Alençon

**Affiliations:** 1 DGIMI, Univ Montpellier, INRAE, Montpellier, France; 2 INRAE, UMR-IGEPP, BioInformatics Platform for Agroecosystems Arthropods, Campus Beaulieu, Rennes, France; 3 INRIA, IRISA, GenOuest Core Facility, Campus de Beaulieu, Rennes, France

**Keywords:** Divergent selection, Fall armyworm, Genome hitchhiking, Genomic differentiation, Genome-wide congealing, Speciation, Speciation continuum, *Spodoptera frugiperda*

## Abstract

**Background:**

The process of speciation involves differentiation of whole genome sequences between a pair of diverging taxa. In the absence of a geographic barrier and in the presence of gene flow, genomic differentiation may occur when the homogenizing effect of recombination is overcome across the whole genome. The fall armyworm is observed as two sympatric strains with different host–plant preferences across the entire habitat. These two strains exhibit a very low level of genetic differentiation across the whole genome, suggesting that genomic differentiation occurred at an early stage of speciation. In this study, we aim at identifying critical evolutionary forces responsible for genomic differentiation in the fall armyworm.

**Results:**

These two strains exhibit a low level of genomic differentiation (F_ST_ = 0.0174), while 99.2% of 200 kb windows have genetically differentiated sequences (F_ST_ > 0). We found that the combined effect of mild positive selection and genetic linkage to selectively targeted loci are responsible for the genomic differentiation. However, a single event of very strong positive selection appears not to be responsible for genomic differentiation. The contribution of chromosomal inversions or tight genetic linkage among positively selected loci causing reproductive barriers is not supported by our data. Phylogenetic analysis shows that the genomic differentiation occurred by sub-setting of genetic variants in one strain from the other.

**Conclusions:**

From these results, we concluded that genomic differentiation may occur at the early stage of a speciation process in the fall armyworm and that mild positive selection targeting many loci alone is sufficient evolutionary force for generating the pattern of genomic differentiation. This genomic differentiation may provide a condition for accelerated genomic differentiation by synergistic effects among linkage disequilibrium generated by following events of positive selection. Our study highlights genomic differentiation as a key evolutionary factor connecting positive selection to divergent selection.

## Background

The process of speciation is initiated from the genetic differentiation of a few loci and then continued by the enlargement of differentiated loci to a whole genome sequence [1]. If a geographic barrier interrupts the gene flow between a pair of diverging populations, genome differentiation may occur readily through the accumulation of mutations by selection or drift. Here, we define genomic differentiation (GD) as a status in which the vast majority of genomic sequences (e.g., > 90%, which was arbitrary chosen in this study) has a significant proportion of variance in genetic differences explained by the genetic difference between multiple diverging taxa, as defined by Weir and Cockerham’s F_ST_ [2]. However, if such geographic barriers do not exist, thus gene flow may occur relatively easily, then GD is impeded by gene flow between a pair of diverging populations because recombination in hybrids constantly homogenizes the DNA sequences [3]. Therefore, a special condition is necessary for GD to overcome this homogenizing effect of gene flow.

Several speciation models involving this special condition have been proposed to explain GD in the presence of gene flow. For example, in the presence of very strong population-specific positive selection (i.e., when selection coefficient is higher than migration rate [4] or recombination rate [5]), the diverging effect of selection is stronger than the homogenizing effect of gene flow, and GD can be generated (Additional file 1: Fig. S1A). Alternatively, instead of a single event of strong positive selection, if many loci are targeted by mild positive selection, the combined effect of selection can be sufficiently strong to reduce the migration rate across the whole genome, enabling GD [6, 7] (termed genome hitchhiking model [8]) (Additional file 1: Fig. S1B). If selective sweeps target a very large number of loci and the average distance from a locus to a nearest selectively targeted locus decreases, then GD might occur by genetic linkage to the targets [9] (Additional file 1: Fig. S1C). Certain genome structures may contribute to the GD as well. For example, if positively selected loci causing reproductive isolation are tightly genetically linked, a long DNA sequence containing these loci can be differentiated up to several Mb [10, 11] (Additional file 1: Fig. S1D). Alternatively, if a positively selected target has a close genetic distance (the frequency of recombination events between loci) to chromosomal rearrangement, a long DNA sequence can be differentiated as well (Additional file 1: Fig. S1E) [12, 13].

The contribution of genome structures to GD has been empirically supported in a wide range of species, involving chromosomal rearrangements [10, 14–18] or tight genetic linkage among selectively targeted loci causing reproductive barrier [12, 19, 20]. In these cases, genetically differentiated elements are located within a single linkage disequilibrium, and these elements are effectively fixed in a population as a single locus. Therefore, the genetic differentiation of completely unlinked loci (such as different chromosomes) remains unexplained. Empirical supports for genome hitchhiking models have been presented from positive correlations between the genetic differentiation at neutral loci and ecological adaptive differences rather than the geographic distance [21] (termed isolation by adaptation [22, 23]). In this explanation, genetic differentiation at neutral loci is not a direct consequence of divergent selection. Instead, this differentiation is a consequence of the genomic and combined effect of selection targeting many loci. Although genome hitchhiking is expected to generate this positive correlation, it is still unclear whether genome hitchhiking initiates or reinforces genetic differentiation in cases of isolation by adaptation. In short, the cause of GD during the speciation process is still largely unknown.

The fall armyworm, *Spodoptera frugiperda* (Lepidoptera: Noctuidae), is native in South and North America. Since 2016, fall armyworms have invaded Sub-Sahara Africa, India, South East Asia, East Asia, and were recently detected in Egypt and Australia (https://www.cabi.org/isc/datasheet/29810). The fall armyworm exists as two sympatric strains, corn strain (sfC) and rice strain (sfR), named after their preferred host-plants, throughout their entire habitats [24]. The proportion of hybrids between these two strains can reach 16% [25], implying frequent gene flow. These two strains have different mating time and sexual pheromone blends [26–28], suggesting pre-mating reproductive isolation. Hybrids generated in our lab have reduced fitness compared with parental strains [29], implying the existence of post-zygotic reproductive isolation. Reciprocal transplant experiments show differential fitness between original and alternative hosts in these two strains [30], suggesting differentiated ecological niches. The existence of reproductive isolation and differentiated ecological niches implies that these two strains are in the process of speciation.

In a previous study, we observed that the two strains collected in Mississippi exhibit GD with a statistical significance, while the extent is very low (average F_ST_ = 0.019) [31]. This pattern of genetic differentiation is potentially a snapshot that two diverging groups just entered the phase of GD at an early stage of speciation. This low level of GD makes the fall armyworm an ideal model species to pinpoint minimal evolutionary forces responsible for GD. This pattern of GD can be generated by a single event of very strong positive selection (for example, insecticide resistance), by mild positive selection targeting many loci, or by the genomic structure of fall armyworms (i.e., chromosomal rearrangement, or by tight genetic linkage among positively selected loci causing reproductive isolation). In this study, we aim at identifying minimal evolutionary forces responsible for GD at the early stage of speciation in the fall armyworm based on population genomics approach. We tested the role of strong or mild positive selection in GD. We also tested the contribution of genome structures to GD. Finally, we inferred an evolutionary history toward GD.

## Results

### Genome assembly and variant identification

Since the existing reference genome assemblies by Gouin et al. are fragmented [31], we generated a new genome assembly using 33.1X PacBio reads from sfR. The resulting assembly is 380 Mb in size (Table 1). This size is closer to the expectation by flow cytometry (396 ± 3 Mb) [31] than the assemblies by Gouin et al. (438 Mb and 371 Mb for sfC and sfR, respectively). N50 of our assembly is 1129 kb, while that of Gouin et al. is 52.8 kb and 28.5 kb for sfC and sfR, respectively, implying increased contiguity. The new assembly has a higher proportion of complete BUSCO (Benchmarking Universal Single-Copy Orthologs) genes [32] (1616/1658 = 97.4%) than Gouin et al. (1461/1658 = 88.1% and 1551/1658 = 93.5% for sfC and sfR, respectively), implying increased correctness. We identified 22,026 protein-coding genes in the genome assembly. BUSCO analysis shows that the correctness of gene annotation was increased as well (Additional file 19: Table S1).

**Table 1:** The comparison of the reference genome assemblies

Statistics	Liu et al.		Gouin et al.		This study
	Male	Female	sfC	sfR	
Contiguity					
Assembly size (bp)	543,659,128	531,931,622	437,876,304	371,020,040	379,902,278
Number of sequences	21,840	27,258	41,557	29,127	1054
N50 (bp)	14,162,803	13,967,093	52,781	28,526	1,129,192
L50	16	17	1616	3761	91
N90 (bp)	6440	5122	3545	6422	165,330
L90	3030	5612	18,789	13,881	421
Correctness (BUSCO)					
Complete	1576	1577	1461	1551	1616
Complete and single-copy	1442	1480	1276	1518	1573
Complete and duplicated	134	97	185	33	43
Fragmented	45	48	127	69	11
Missing	37	33	70	38	31
Total	1658	1658	1658	1658	1658

Recently, Liu et al. generated two genome assemblies from a natural invasive population of the fall armyworm [33]. These two assemblies have higher contiguity, estimated from N50 and the number of sequences, than the assemblies generated in this study (Table 1). However, the assembly sizes are far larger than the expectation by flow-cytometry (532–544 Mb and 396 ± 3 Mb for Liu et al. and the expected size [31], respectively), perhaps due to heterozygous positions or due to different genome assembling approaches. These two assemblies have lower numbers of complete and single-copy BUSCO genes (1442 and 1480) than our assembly (1573), demonstrating lower correctness than ours. The assemblies by Liu et al. have higher numbers of ‘complete and *duplicated*’ BUSCO gene (134 and 97) than ours (43), suggesting that misassemblies may inflate the size of the genome assemblies.

Resequencing data from 18 wild-caught females (nine sfC and nine sfR) obtained from our previous study [31] were mapped against the reference genome assembly. One individual from sfR was excluded in the following analysis because of a particularly low average read depth (< 15X coverage) (denoted as R1, Gouin et al. [31]) (Additional file 2: Fig. S2). After variant calling, we performed very strict filtering (see *Variant calling* subsection in Methods). The remaining callable positions are 205,381,292 bp, implying that the filtering discarded ~ 175 Mb positions from the assembly. The number of identified single nucleotide polymorphisms (SNPs) is 49,832,320.

### GD between sfC and sfR

**Fig. 1:**
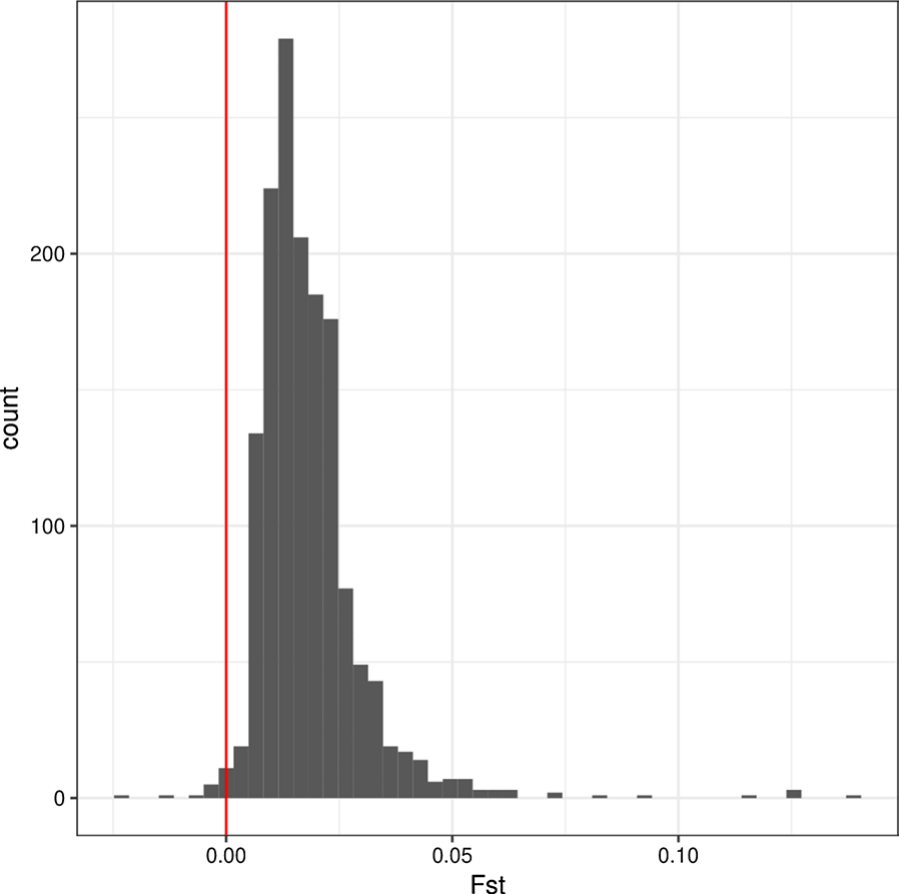
Whole genome differentiation between strains The histogram was made from F_ST_ calculated in 200 kb windows. The red vertical bar indicates F_ST_ = 0

The Weir and Cockerham’s weighted mean F_ST_ [2] between sfC and sfR from the whole genome sequence is 0.0174, which is comparable to our previous study (0.019) [31]. As this low level of differentiation could be caused by chance, we calculated F_ST_ from two groups generated by randomization with 500 replications. We observed that no randomized grouping has higher F_ST_ than the grouping according to strains (equivalent to p-value < 0.002), implying a significant genetic differentiation between strain. This low level of genetic differentiation can be either caused by many loci with low levels of differentiation or by a few loci with very high levels of differentiation. To test these two possibilities, we calculated F_ST_ in 200 kb windows, and we observed that 99.2% of the total windows have F_ST_ greater than 0 (Fig. 1). The statistical significance of genomic differentiation was tested for each untruncated 200 kb window, followed by multiple testing correction. These untruncated 200 kb windows correspond to 79.15% of the genome assembly. We observed that 93.8% of windows have statistical significance inferred by the comparison of F_ST_ between the groupings according to the strains and random groupings (FDR-corrected p-values < 0.20). As the proportion of genetically differentiated windows might be affected by window sizes, we re-calculated F_ST_ from 10 kb windows, observing that 91.12% of windows have F_ST_ higher than 0. These results demonstrate the existence of GD between sfC and sfR.

### The role of positive selection in GD

**Fig. 2:**
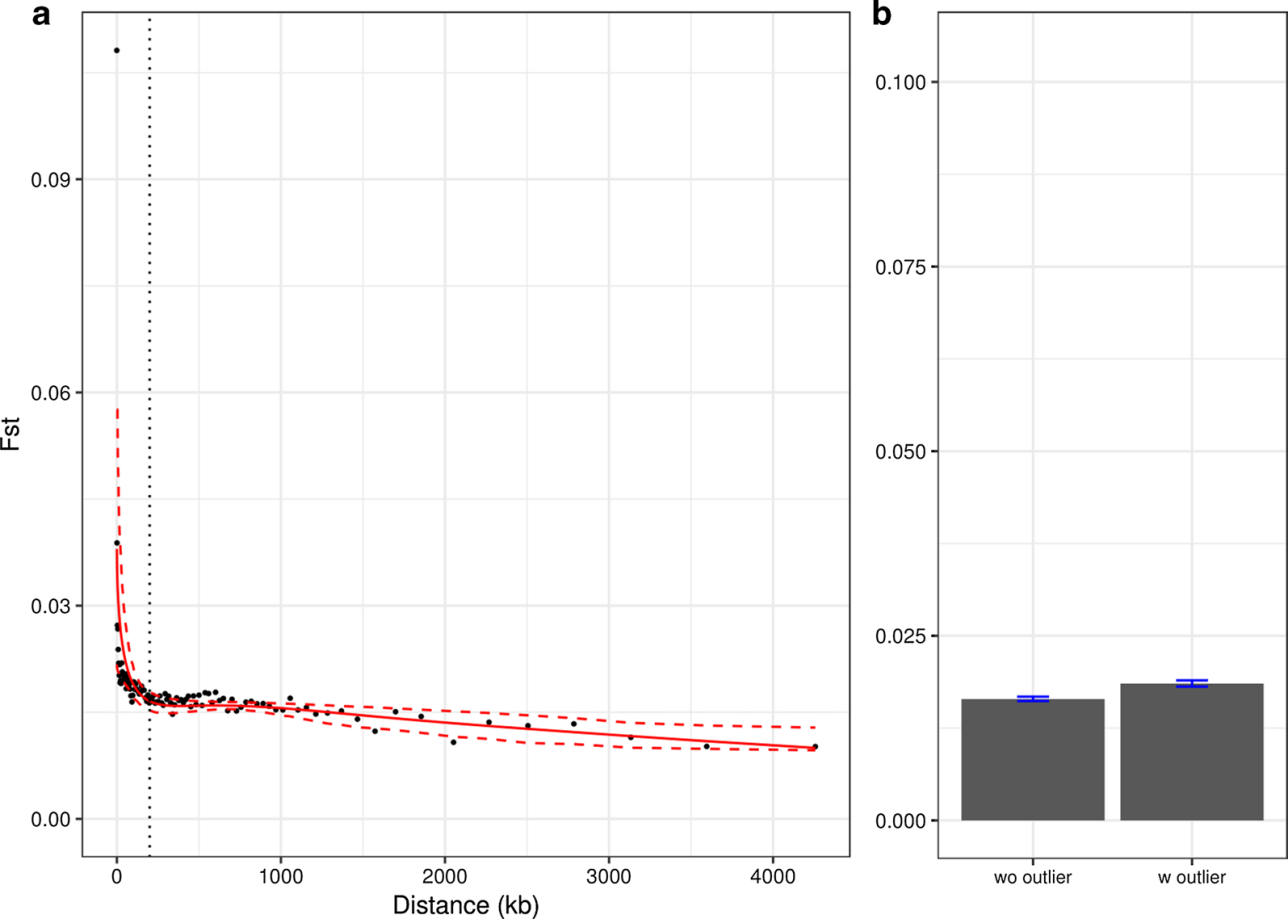
The effect of genetic linkage to selectively targeted loci on F_ST_. **a** F_ST_ calculated according to the distance from each nucleotide in the assembly to the nearest outlier of genetic differentiation. The left-most point corresponds to F_ST_ from the outliers. The solid red curve is fitted by smooth-spline with degree of freedom = 4, and the red dotted curves are 95% confidence intervals with 1000 bootstrapping. The vertical dotted line indicates the distance equal to 200 kb. **b** The barplot shows F_ST_ from the scaffolds with outliers and without outliers. The error bars indicate 95% bootstrapping confidence intervals calculated from the resampling from 100 kb windows with 1000 replications

To investigate the role of selection in GD, we identified genomic footprints of selective sweeps, a reduction in genetic diversity at linked sites to a target of positive selection by hitchhiking effects [34]. These footprints are expected to have higher levels of genetic differentiation because coalescence time is proportional to local effective population sizes. Thus, we identified the outliers of genetic differentiation as targets of selective sweeps (See box1 at [35]). To calculate the level of genetic differentiation between sfC and sfR, we used hapFLK scores, which represent correlated haplotypes according to pre-determined groups [36]. Using the hapFLK scores to identify outliers has an advantage over F_ST_, because the influence of demographic history can be effectively controlled and because statistical dependence among SNPs within linkage disequilibrium is considered. If each minimum of 100 consecutive SNPs in a minimum of 1 kb has a significantly higher hapFLK score than the rest of the genome (*p* < 0.001), we defined this locus as an outlier. In total, 423 outliers were identified from 148 scaffolds out of 1,054, and the length is 4095 bp on average. The averaged F_ST_ among all outliers is 0.108 (the leftmost point at Fig. 2a), which is much higher than the genomic average (0.0174).

We tested the effect of genetic linkage to the targets of positive selection. If genetic linkage contributes to the genetic differentiation, F_ST_ is expected to be higher in the vicinity of the targets. We calculated the distance from each nucleotide of the reference genome assembly to the nearest outliers and sorted the nucleotides according to the distance. Then, 100 groups were generated according to the distance in a way that each group has comparable numbers of nucleotides, followed by calculating F_ST_ from each group. The non-linear regression curve generated by the smooth-spline shows that, if the distance is less than 200 kb, F_ST_ decreases rapidly as the distance increases (Fig. 2a). If the distance is higher than 200 kb, on the other hand, the change in F_ST_ is negligible. This result shows that the effect of genetic linkage to selectively targeted sites is clearly observed only when the distance is shorter than 200 kb. The total length of sequences within this distance (< 200 kb) is 83,103,350 bp, which corresponds to 21.9% of the genome assembly.

The F_ST_ calculated along the physical distance from the nearest outlier is always greater than 0.0102, even when the distance is higher than 2 Mb. F_ST_ calculated from scaffolds without outliers is 0.0164, which is significantly lower than that from scaffolds with outliers (F_ST_ = 0.0185; p-values < 0.01, bootstrapping test) but higher than zero (Fig. 2b). This result indicates that the genetic linkage to positively selected sites is not sufficient for GD because genetic differentiation is also observed from loci far from the outliers (e.g., > 2 Mb in the distance) and the scaffolds without outliers. The genetic differentiation at loci unlinked to the target of positive selection can be explained by the genome hitchhiking model [6–8], which suggests that the combined effect of mild positive selection caused GD regardless of genetic linkage to the targets.

An alternative explanation for the observed pattern of GD is a single event of very strong positive selection, rather than multiple events of mild positive selection. In this case, the vast majority of genomic positions are included in a single cline generated by a single event of very strong positive selection [5]. Then, the contribution of the other mild positive selection could be negligible rather than a cause of GD. Therefore, we tested whether a single event of population-specific positive selection sufficiently strong to create GD is supported. For this test, we estimated selection coefficient (*s*) required for GD from a theoretical prediction from the classical theory of hitchhiking effect (Eq. 8 in [34]). We assumed that the reduction in π is a signature of increased genetic differentiation by the hitchhiking effect of population-specific positive selection because coalescence time is proportional to the local population size. First, we estimated the effective population size of the fall armyworm for this test. The average π from sfC and sfR is 0.0435 (0.0434–0.0437 of 95% confidence interval calculated by bootstrapping with 1000 replications) and 0.0443 (0.0441–0.0445 of 95% confidence interval). Based on the assumption that mutation rate is the same with Lepidoptera *Heliconius melpomene *[37], the estimated population sizes of sfC and sfR are 0.0435/(4 × 2.9 × 10^–9^) = 3.75 × 10^6^ and 0.0443/(4 × 2.9 × 10^–9^) = 3.81 × 10^6^, respectively (please note that H = 4 × Ne × μ, where H, Ne, and μ are heterozygosity, effective population size, and mutation rate, respectively). Second, we estimated the required *s* for GD with these effective population sizes. When *s* is within a biologically realistic range (0 < *s* ≤ 1), reduction in π was observed only when the genetic distance is less than 20 cM, implying that GD is not observed (Additional file 3: Fig. S3A). Then, we performed the calculation with a biologically unrealistic range of *s* (5 ≤ *s* ≤ 100). A reduction in π was observed at all linked loci (0–100 cM) only when *s* is higher than 20 (Additional file 3: Fig. S3B). This result implies that GD can be created only by biologically unrealistic positive selection, unsupporting the hypothesis that GD was generated by a single event of very strong positive selection.

**Fig. 3:**
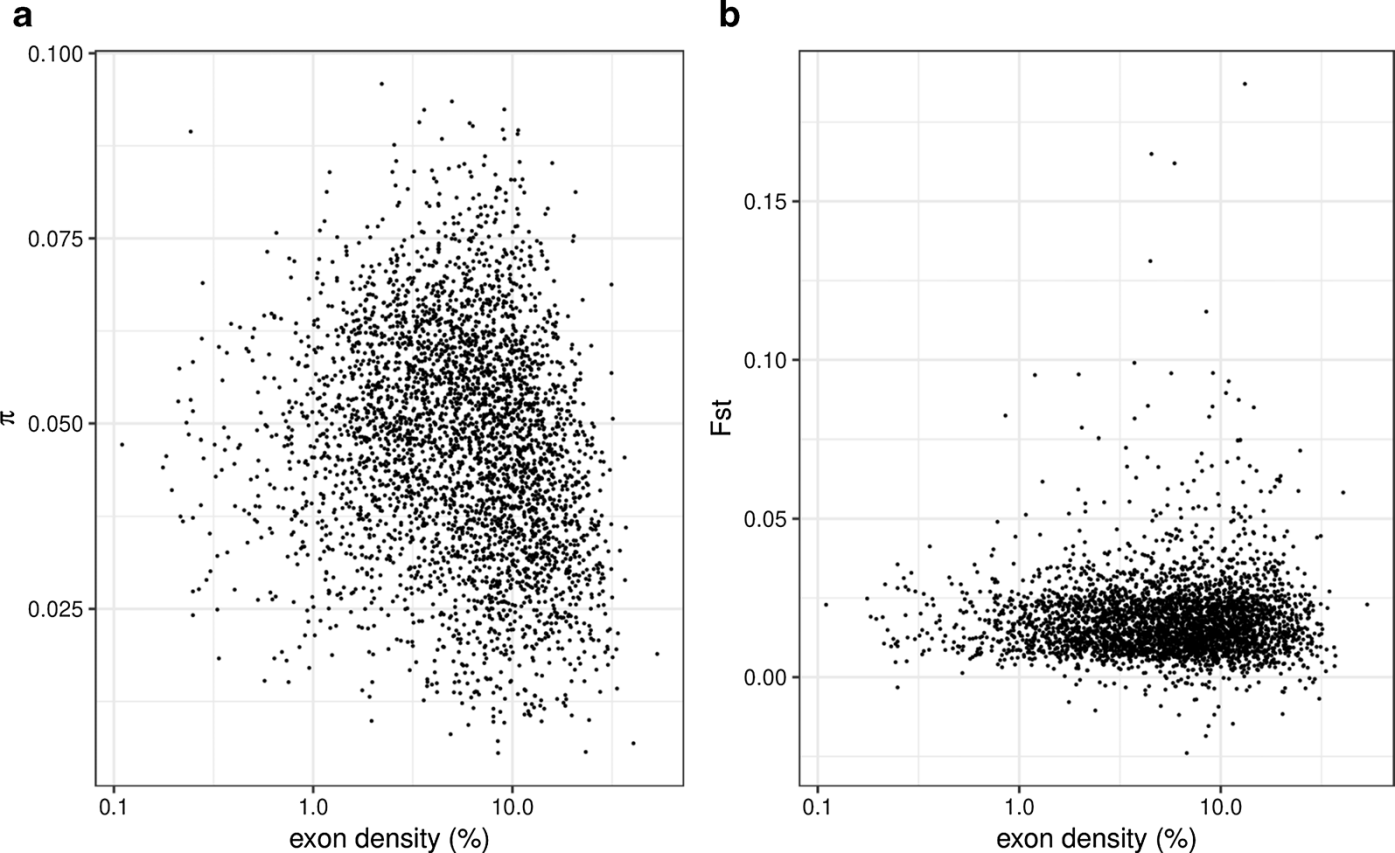
Testing background selection The relationship of exon density with π (**a**) and F_ST_ (**b**)

An alternative explanation for the observed negative correlation between F_ST_ and the distance from outliers (Fig. 2a) is a predominant role of background selection [38] in generating the pattern of F_ST_. In other words, the negative correlation between F_ST_ and the distance could be a byproduct of a correlation between F_ST_ and the strength of background selection, rather than selective sweeps. Background selection may target all selectively constrained sequences in principle, while the strength is proportional to the rate of deleterious mutation [39]. If outliers have a higher proportion of selectively constrained sites than the rest of the genome, background selection alone could generate the observed negative correlation between F_ST_ and the distance from outliers. Thus, we tested if background selection generates the observed negative correlation between the distance and F_ST_. As it is reasonable to assume that exons have a higher deleterious mutation rate than non-exonic sequences [40], we used the exon density (a proportion of exonic nucleotides in a window) as a proxy of the strength of background selection. The exon density calculated in 100 kb windows is negatively correlated with π (Spearman’s ρ = − 0.214, p-value < 2.2 × 10^–16^) (Fig. 3a), showing that the strength of background selection is positively correlated with the exon density. F_ST_, however, is not significantly correlated with exon density (ρ = 0.021, p-value = 0.2032) (Fig. 3b). The same trend was observed when we calculated the correlations from scaffolds without outliers (Additional file 4: Fig. S4). Exons are overrepresented at the outliers compared with non-outliers with statistical non-significance (odd ratio = 1.109; p-value = 0.091, randomization test with 1,000 replications) implying that the outliers may not even have experienced stronger background selection than the rest of genomic loci. This result demonstrates that background selection is not a dominating factor determining the local level of genetic differentiation. Thus, it is unlikely that background selection alone caused the observed positive correlation between F_ST_ and distance to the outliers.

### The role of genome structures

According to the divergence hitchhiking model [12, 13], tight genetic linkage among selectively targeted loci within a genome is the main cause of GD. If a locus is targeted by strong positive selection, then the effective rate of migration is reduced in this region, and following events of positive selection targeting sequences within this region may generate a long stretch of differentiated DNA up to several Mb in length. In this case, a long sequence with genetic differentiation is expected. The longest outlier is 33,110 bp, which corresponds to 0.0087% of the genome. Therefore, a significant contribution of a single locus to GD is unlikely. The length of outliers can be underestimated by the fragmented genome assembly if the longest outliers are generally observed at the end of a scaffold. Thus, we analyzed the distribution of hapFLK scores along the scaffolds with ten longest outliers. All the ten outliers were found in the middle of the scaffolds, with the exception of one outlier on Contig306 (Additional file 5: Fig. S5). Therefore, an outlier with much longer than 33,110 bp is not likely to exist.

We investigated the role of strain-specific chromosomal inversions from mappings of the resequencing data against the reference genome. If a chromosomal inversion is strain-specific, then the difference in the allele frequency of the chromosomal inversions is expected to be 1. We identified 1227 loci with chromosomal inversions, 1071 bp in median length. The majority of loci with chromosomal inversions are shorter than 10 kb (1101/1227 = 89.73%), while the 126 and 44 chromosomal inversions are longer than 10 kb and 100 kb, respectively. Among these loci, the highest difference in the allele frequency is 0.667, implying that strain-specific chromosomal inversion is not observed. As the reference genome assembly used in this study is still fragmented, a proportion of long chromosomal inversions may remain unidentified. Therefore, we also identified chromosomal inversions that could be identified as translocation between scaffolds. The number of identified inter-scaffold translocation is 1,644, and the highest difference in the allele frequency is 0.625. This result shows that strain-specific chromosomal inversion is not observed. Therefore, it is unlikely that chromosomal inversions contribute to GD.

### From gene to genome differentiation

Then, we inferred the evolutionary history from the genetic differentiation of a few loci to GD between sfC and sfR. If selection targeting an outlier initiates genetic differentiation, this outlier is expected to have a higher level of absolute genetic differentiation, which can be estimated from the d_XY_ statistics, than the genomic average due to a more ancient divergence time [41]. Nine out of the 423 outliers have higher d_XY_ than the genomic average (FDR corrected p-value < 0.05) (Additional file 6: Fig. S6). These outliers contain four protein-coding genes, including NPRL2 and Glutamine synthetase 2. NPRL2 is a down-regulator of TORC1 activity, and this down-regulation is essential in maintaining female fecundity during oogenesis in response to amino-acid starvation in Drosophila [42]. Glutamine synthetase 2 is important in activating the TOR pathway, which is the main regulator of cell growth in response to environmental changes to maintain fecundity in planthoppers [43]. The ancient genetic differentiation of these female fecundity genes raises the possibility that disruptive selection on female fecundity might be responsible for initiating genetic differentiation between strains. The function of the remaining two genes is unclear.

The rest of the outliers have lower π than the genomic average in both strains (Additional file 7: Fig. S7), in line with selective sweeps. The d_XY_ calculated from these outliers is lower than the genomic average (Additional file 8: Fig. S8), showing that the outliers have a shorter coalescence time than the genomic average, which is expected in the presence of selective sweeps (see below for the possibility of background selection).

The outliers contain 297 protein-coding genes (Additional file 20: Table S2). These protein-coding genes include a wide range of genes important for the interaction with host-plants, such as P450, chemosensory genes, immunity gene, oxidative stress genes, esterase, and serine protease [31] (Additional file 19: Table S3). This result shows that positive selection on these host-plant genes potentially contributed to GD.

### GD by subsetting variants

**Fig. 4:**
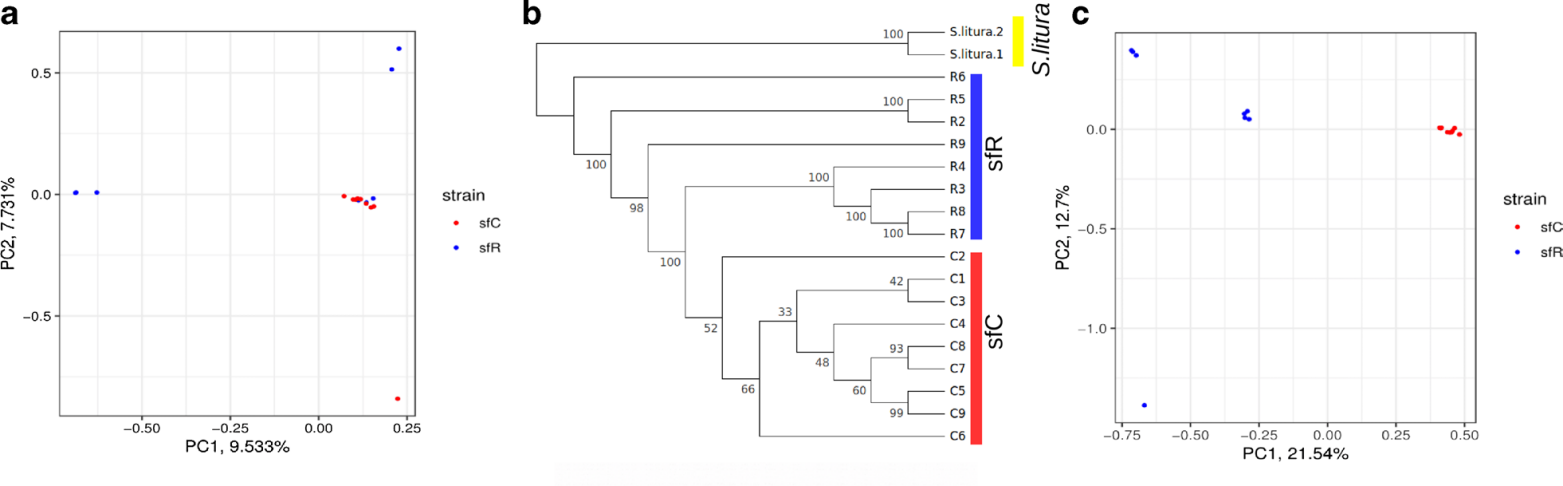
Subsetting of sfC variants from ancestral sfR variants. **a** Principal component analysis from the whole genome. The red and blue dots represent sfC and sfR, respectively. **b** Phylogenetic tree reconstructed from the whole genome sequences. **c** Principal component analysis from the outliers

We inferred the genetic relationship among individuals of sfC and sfR. The principal component analysis shows that the genetic variation of sfR includes that of sfC (Fig. 4a). This result suggests the possibility that sfR is an ancestral status and that sfC was derived from the sfR. To test this possibility further, we reconstructed a BIONJ phylogenetic tree from whole genome sequences with *S. litura* as an outgroup. The resulting tree shows that sfC individuals constitute a monophyletic group with a moderate level of bootstrapping support value (52%) while clades containing sfC and a subset of sfR have a high bootstrapping supports (98–100%) (Fig. 4b). A k-mer-based phylogenetic tree also shows that sfC individuals constitute a monophyletic group within the sfR clade (Additional file 9: Fig. S9). These phylogenetic trees support the hypothesis that sfC was derived from ancestral sfR. However, it should be noted that gene flow between sfC and sfR might affect the phylogenetic pattern. The genetic relationship among sfC and sfR individuals was also analyzed from the ancestry coefficient, and distinct origins of sfC and sfR are unsupported (Additional file 10: Fig. S10). This result can be interpreted in a way that positive selection targeting the outliers causes the GD by sub-setting sfC variants from ancestral sfR variants.

In principle, background selection also increases the level of genetic differentiation because of reduced coalescent time, particularly if the rate of recombination is very low. If background selection is responsible for the identified outliers, the pattern of grouping is expected to be qualitatively the same between outliers and whole genome sequences, while the level of genetic differentiation is quantitatively different. Principal component analysis exhibits a clear grouping according to the strains at the outliers (Fig. 4c). This pattern differs from whole genome sequences, which shows that sfC is a sub-group of sfR (Fig. 4a). Therefore, background selection is unlikely to be responsible for the outliers. Instead, the result can be explained by population-specific positive selection, which generated differentiated whole genome sequences from an original population by the fixation of newly generated beneficial mutations.

The different patterns from the principal component analysis between the outliers and whole genome sequences could be due to the low quality of genotypes at outliers. To test this possibility, we compared variant calling scores between outliers and the rest of genetic variants. We observed that variants at outliers have higher variant calling scores (747.05 on average) than the rest variants (617.07) (p-value < 0.001, bootstrapping test with 1,000 replications). Therefore, it is unlikely that SNPs at outliers have a higher rate of false-positive positive than the genomic average. Please note that the expected proportion of false positives at outliers is 10^(−747.05/10)^ = 1.97 × 10^–75^.

We tested the possibility of an extreme case of where both sfC and sfR have monophyly, and these two strains are a sister group of each other, but all identified sfR individuals except R6 in Fig. 4b are F1 hybrids between sfR females and sfC males. Then, our conclusion that sfC was derived from sfR could be misled by massive hybridization between strains. If this possibility is true, maternally-derived mitochondrial CO1 genes used to identify strains in this study [31] will have distinctly different sequences between R2–R9 and C1–C9, while paternally derived sequences will not show such a pattern. As all individuals analyzed in this study are females, the Z chromosomes were derived from males in the parental generation (please note that Lepidoptera has ZZ males and ZW females). If all identified sfR individuals except R6 are F1 hybrids, genetic differentiation between sfC and sfR except for R6 is not expected to be observed from Z chromosomes. TPI gene is known to be linked to Z chromosomes in the fall armyworm [44]. The scaffold containing TPI gene is 4,826,061 bp in length, and the number of SNPs is 308,810. The F_ST_ calculated between sfC and sfR without R6 is 0.056, which is higher than the genomic average (0.0174). We calculated F_ST_ from randomized groupings with 500 replicates, and only two replicates have F_ST_ higher than 0.056, corresponding to the p-value equal to 0.004. This result demonstrates a significant genetic differentiation of paternally derived Z chromosomes between strains. Therefore, we exclude the possibility of the extreme case with F1 hybrids.

### Mitochondrial differentiation

Our observation that sfC was derived from sfR is different from previous reports, which show that sfC and sfR are sister groups between each other across the wide range of geographic populations based on the mitochondrial DNA [45, 46]. From our data, the mitochondrial genomes have almost completely differentiated between strains (F_ST_ = 0.920), and sfC and sfR appear to be the sister group of each other (Additional file 11: Fig. S11—Additional file 13: S13), confirming the previous reports. A molecular clock study shows that the mitochondrial genomes diverged between sfC and sfR two million years ago [45], which corresponds to 2 × 10^7^ generations according to the observation from our insectarium (10 generations per year). Results from forward simulation show that the nuclear F_ST_ (0.0174) is unlikely to be generated during this divergence time with a wide range of migration rates (Additional file 14: Fig. S14), suggesting that the mitochondrial genome diverged more anciently than the nuclear genome.

## Discussion

In this paper, we show in fall armyworms that mild positive selection contributed to GD at the early stage of the speciation process by combined effects of mild positive selection targeting many loci. Linkage to positively selected sites also contributes to GD by increasing genetic differentiation in ~ 21.9% of the genome. We do not find the contribution of very strong positive selection or genomic structures in GD, suggesting that mild positive selection targeting many loci appears to be sufficient for generating a pattern of GD at the early stages of the speciation process.

**Fig. 5:**
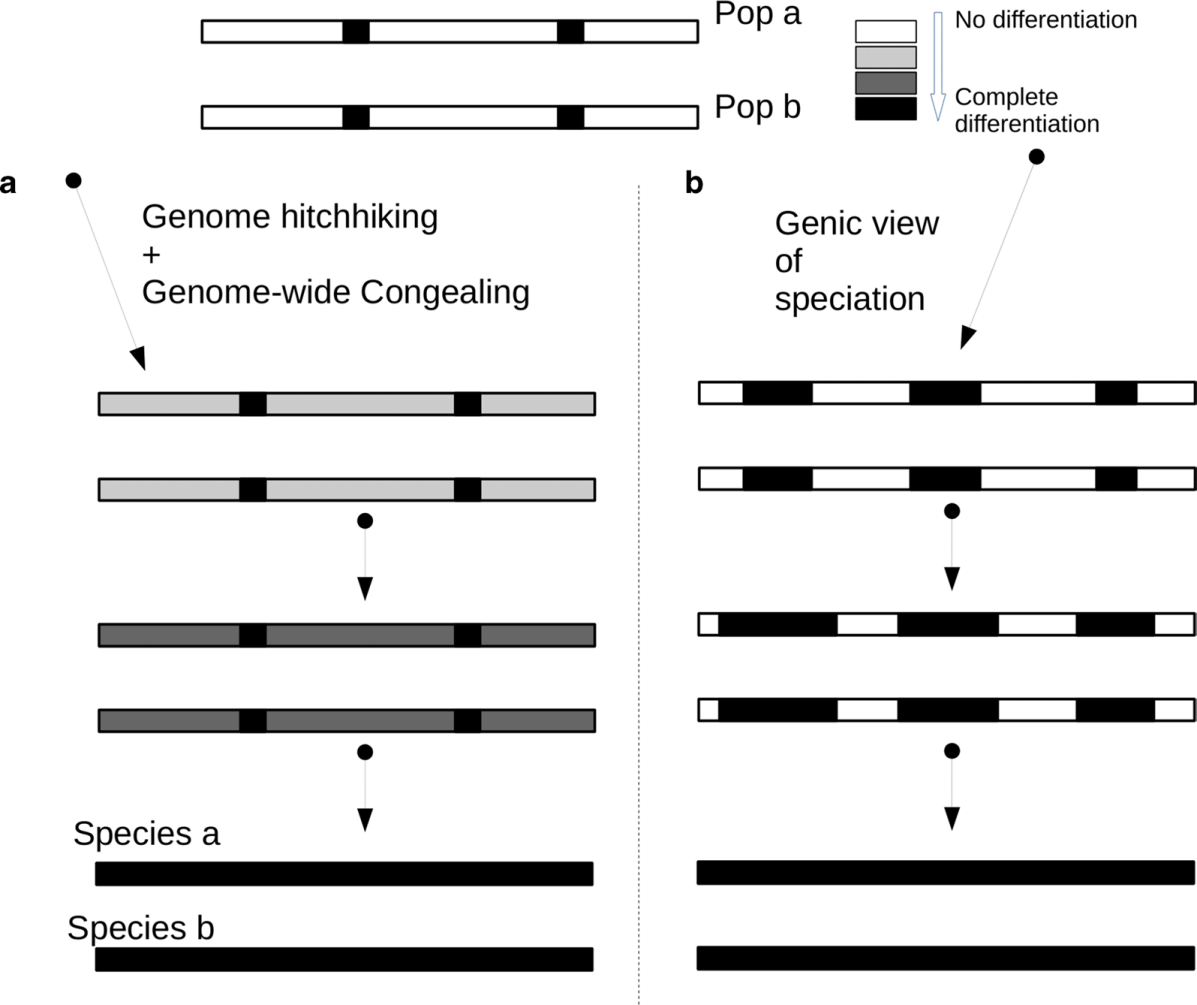
Two speciation models concerning whole genome differentiation The process of speciation initiates from genetic differentiation between population a and population b, and finishes when these two populations are evolved to species a and species b with completely differentiated genomes. **a** According to the genome hitchhiking and the genome-wide congealing models [8, 48], divergent positive selection targeting many loci causes whole genome differentiation with a low extent by the combined effect of mild positive selection. Following divergent positive selection rapidly accelerates the rate of whole genome differentiation by the synergistic effect of linkage disequilibrium across the whole genome until whole genome sequences are completely differentiated. In this model, whole genome differentiation is generated at the early stage of a speciation process. **b** According to the genic view of speciation [1], the fully differentiated loci are progressively enlarged or additional fully differentiate loci are generated until whole genome sequences are differentiated. In this model, whole genome differentiation is generated at the end of a speciation process

Once a GD pattern is generated, the rate of genetic differentiation across the whole genome can be accelerated by following positive selection until the end of the speciation process (Fig. 5a). Theoretical studies [4, 47] suggest that the level of GD has a non-linear relationship with a number of accumulated selectively targeted loci. As positive selection generates linkage disequilibrium at the targeted locus, multiple events of population-specific positive selection will generate linkage disequilibrium according to the populations. If the number of targets is higher than a certain threshold, targeted loci have a synergistic effect in increasing linkage disequilibrium among targets, consequently resulting in the acceleration of GD. The non-linear dynamics were termed genome-wide congealing [48]. The GD between sfC and sfR implies that linkage disequilibrium has been generated across the whole genomes, raising the possibility that any following positive selection may accelerate the GD by the synergistic effects of linkage disequilibrium, in line with the genome-wide congealing model. In this case, positive selection acts on a pair of diverging taxa as divergent selection because positive selection contributes to the process of speciation through GD. In other words, positive selection alone may be sufficient for speciation during the entire process of speciation, first by generating GD due to the combined effect of mild positive selection and genetic linkage to selectively targeted sites, as shown in this paper, and second by accelerating GD by the synergistic effect of linkage disequilibrium at selectively targeted loci. We do not argue that sfC and sfR will evolve into two species in the future. Instead, we argue that a condition for the accelerating GD is made in these two strains by following events of positive selection. This explanation is in contrast with the ‘genic view of speciation’ [1] (Fig. 5b). In this model, speciation is initiated from the differentiation from a few loci, and continued by the enlargement of differentiated loci, and finished when the pattern of GD is generated. According to this model, GD is passively generated at the end of a speciation process.

Reports on reproductive isolation between sfC and sfR are contradictory. A group of studies reported that hybrids from female sfC and male sfR have lower fitness or reproductive success than that from female sfR and male sfC [25, 29, 49, 50]. On the other hand, another group of studies reported the opposite pattern [51–53]. From our conclusion that sfC was derived from sfR, we raise the possibility that the heterogeneous genetic background of sfR caused these contrasting patterns. If sfC was derived from sfR, the genetic distance between sfC and sfR individuals is different according to the used sfR individuals. Then, the pattern of fitness reduction in hybrids can also be different depending on the used sfR individuals, and even opposite patterns might be generated.

We observed a discrepancy in the phylogenetic pattern between nuclear and mitochondrial genomes. This discrepancy could be explained by the initiation of genetic differentiation promoted by the disruptive selection on a female fecundity gene (see *from gene to genome differentiation* subsection in Results) and the ancient genetic differentiation of mitochondrial genomes (see “Mitochondrial differentiation” subsection in “Results”). We observed that the genetic differentiation between strains was initiated from a female fecundity gene. If the genetic differentiation between sfC and sfR was initiated by disruptive selection on female fecundity, maternal genealogy would show a bifurcating genetic transmission pattern between strains. However, the rest of the nuclear genome is expected to have more recent differentiation than the female fecundity genes because gene flow mixed the other nuclear sequences between the strains. As mitochondria are inherited through maternal lineage, the mitochondrial sequences will show the same bifurcating genetic transmission pattern as the female fecundity gene. Then, mitochondrial genomes will be differentiated more anciently than nuclear genomes. This explanation does not exclude the possibility of direct positive selection on mitochondrial genes.

Several genetic markers have been proposed to identify strains, including mitochondrial CO1 [54], FR elements (tandem repeats of a 189 bp sequence specific to sfR) [55], and Z-linked TPI [44]. We found that FR elements are a reliable marker to identify strains (Additional file 15: Fig. S15). The concordance of identified strains between mitochondrial CO1 and TPI can be as low as 74% [44]. TPI is included in the gene list within the outliers, suggesting that linked selection is responsible for the differentiation of TPI gene. d_XY_ from TPI (0.0345) is slightly lower than the genomic average (0.0384 with 0.0383–0.0386 of 95% confidence interval), showing that the genetic differentiation of TPI is less ancient than the genomic average. Considering our observation that sfC was derived from sfR, the pattern of genetic variation of TPI gene might differ depending upon the genetic distance between sfC and analyzed sfR individuals (Additional file 16: Fig. S16). Therefore, we propose to use mitochondrial markers for the identification of strains.

The average nucleotide diversity (π) calculated from our resequencing data is 0.0435 and 0.0443 for sfC and sfR, respectively. This π is higher than that of other Lepidopteran species (Additional file 19: Table S4). One of the possible reasons for this high π is the difference in the way of calculating π. Typically, π of the genomic average is calculated from the sum of π at filtered variant positions divided by the assembly size. This method leads to the underestimation of π because all uncalled positions or discarded variants are considered as non-variants. To avoid this underestimation, we calculated the genomic average from the sum of π from filtered variant positions divided by the total number of filtered positions, which include both variants and non-variants. If we calculate the genomic average π by dividing the assembly size, the genomic average π is 0.0235 and 0.0239 for sfC and sfR, respectively, which are within the range of reported cases (Additional file 19: Table S4). However, population explosions of the fall armyworm could increase π as well (Additional file 17: Fig. S17).

We propose a possibility that the probability of speciation could be affected by the size of populations. Species with a large population size may have a higher adaptive evolutionary rate than those with a small population size because larger populations have a higher influx of mutations, of which a proportion has beneficial effects (hard sweeps). In addition, large populations have a higher proportion of existing variants that can be beneficial in the future depending upon environmental changes (soft sweeps) [56]. A positive correlation between adaptive evolutionary rates and population sizes has been reported from a wide range of taxa [57, 58] (but see Galtier [59]). Moreover, a positive correlation between the size of populations and the strength of selective sweeps was reported [60]. If large populations experience a higher frequency of positive selection, species with a large population size might have a higher potential to be speciated because of a higher probability of experiencing accelerated genomic differentiation by synergistic effects among linkage disequilibrium generated positive selection. Thus, we propose a potential link between the taxonomic diversity among species and the genetic diversity within species.

## Conclusions

Here, we showed that mild positive selection is a sufficient evolutionary force for GD at the early stage of speciation in the fall armyworm. A special event of very strong positive selection or special genome structure (i.e., genetic linkage among positively selected loci or genetic linkage between positively selected loci and chromosomal rearrangement) appears not to contribute to GD. This GD may provide a condition for an accelerated genetic differentiation by following positive selection, which causes synergistic effects among the linkage disequilibrium of selectively targeted loci. Therefore, we propose that multiple events of mild positive selection alone are sufficient for speciation and that adaptive evolution alone could be a fuel of species diversity.

The level of GD can be different among different geographical populations in the fall armyworm. Therefore, the fall armyworm can be suitable model species to study the process of GD by providing snapshots of different phases of genetic differentiation, ranging from the initiation of genetic differentiation, the initiation of GD, and the acceleration of GD. Attempts to identify these phases, often termed ‘speciation continuum’, are typically based on closely related multiple species [61, 62]. However, different species may have experienced very different selective pressures. Thus, studying a single species at varying levels of genetic differentiation may shed light on a precise process of GD.

## Methods

### De novo genome assembling

We extracted high molecular weight DNA using the MagAttract© HMW kit (Qiagen) from two sister pupae of sfR raised in our insectarium, with a modification of the original protocol to increase the yield as follows. Initial frozen tissue reduced in powder was split into four tubes, and for each tube we followed the protocol of lysis and affinity purification steps with the volumes and reagents advised by the supplier except at the last elution step, such that we eluted the first tube with 150 µl of Buffer AE and re-used the same 150 µl of buffer AE to elute the three other tubes. During the lysis step, the shaking speed was reduced from 900 to 300 rpm to avoid DNA breakage. The quality of extraction was assessed by checking DNA length (> 50 kb) on 0.7% agarose gel electrophoresis, as well as pulsed-field electrophoresis using the Rotaphor (Biometra) and gel containing 0.75% agarose in 1 × Loening buffer, run for 21 h at 10 °C with an angle range from 120 to 110° and a voltage range from 130 to 90 V. The DNA concentration was estimated by fluorimetry using the QuantiFluor Kit (Promega), which was used to prepare libraries for sequencing. Single-Molecule-Real Time sequencing was performed using a PacBio RSII (Pacific Biosciences) with 12 SMRT cells per strain (P6-C4 chemistry) at the genomic platform Get-PlaGe, Toulouse, France (https://get.genotoul.fr/). The total throughput was 13,259,782,164 bp in 1,692,240 reads. The average read length was 7836 bp.

We newly generated an assembly from Illumina paired-end sequences with 308X coverage from sfR, which was used to generate the sfR assembly in our previous study (Table 1) [31], using platanus 1.2.4 [63]. Then, errors in PacBio reads were corrected by the Illumina assembly using Ectools [64], and uncorrected reads were discarded. The remaining reads were 11,005,855,683 bp in total. We generated a genome assembly with the error-corrected reads using SMARTdenovo [65]. Then, a mapping of the paired-end Illumina reads was performed against this genome assembly using bowtiebowtie2 v2.3.3 [66], and corresponding bam files were generated. We improved the genome assembly with these bam files using pilon v1.22 [67]. Then, we performed scaffolding using BESST v2.2.4 [68]. The gaps were filled using PB-Jelly (version PBSuite_15.8.24) [69]. The correctness of assemblies was assessed using insect BUSCO v3 (insecta_odb9) [32].

### Gene annotation

Protein-coding genes were annotated from the genome sequences using MAKER v2.31.8 [70]. For this annotation, first, repetitive elements were masked using RepeatMasker v4.0.3 [71]. Second, ab initio gene prediction was performed with protein-coding sequences from two strains in *S. frugiperda *[31] and *Helicoverpa armigera* (Harm_1.0, NCBI ID: GCF_002156995), as well as insect protein sequences from *Drosophila melanogaster* (BDGP6) and three Lepidoptera species, *Bombyx mori* (ASM15162v1), *Melitaea cinxia* (MelCinx1.0), and *Danaus plexippus* (Dpv3) in ensemble metazoa. For transcriptome sequences, we used a locally assembled transcriptome from RNA-Seq data from 11 samples using Trinity v2.0.6 [72]. Third, two gene predictors, SNAP v2006-07-28 [73] and Augustus v3.3.1 [74], were trained to improve gene annotations. Multiple trainings of the gene predictors did not decrease Annotation Edit Distance Score. Thus, we used the gene annotation with only one training. Fourth, we discarded all gene predictions if eAED score is higher than 0.5.

### Variant calling

Paired-end Illumina resequencing data from nine female individuals from each of sfC and sfR were obtained from our previous study [31]. We used these Illumina reads to identify variants. Low-quality nucleotides (Phred score < 20) and adapter sequences in the reads were removed using AdapterRemoval v2 [75]. Then, reads were mapped against the reference genome using bowtie2 v2.3.3, with very exhaustive local search parameters (-D 25 -R 5 -N 0 -L 20 -i S,1,0.50), which results in a more exhaustive search than the—very-sensitive parameter preset. Potential PCR or optical duplicates were removed using Picard v1.9 [76]. Variants were called using samtools v1.5 mpileup [77] from the mappings with Phred mapping score higher than 30. Then, we used the called positions if all the following conditions are met; (1) A genotype is determined from all individuals, (2) variant calling score is higher than 40, (3) the read depth is lower or equal to 3200 (which corresponds to around ten times of mean read depth), and (4) higher or equal to 20 (which corresponds to one read per sample). Samtools guideline proposes to discard variant positions if the read depth is higher than twice the average depth (https://github.com/samtools/bcftools/wiki/howto). In the filtered SNP data, the proportion of SNP of which the read depth is higher than twice the average is only 0.32%. Chromosomal rearrangements of resequencing data were inferred from the bam files using BreakDancer v1.3.6 [78].

### Population genetics analysis

We used vcftools v0.1.13 [79] to calculate population genetics statistics, such as π and F_ST_. d_XY_ was calculated using custom perl scripts (available on request). To estimate the genetic relationship among individuals, we first converted the VCF (variant calling format) file to plink format using vcftools v0.1.13, then PCA was performed using flashpca v2 [80]. For ancestry coefficient analysis, we used sNMF v1.2 [81] with K values ranging from 2 to 10, and we chose the K value that generated the lowest cross-entropy.

The outliers of genetic differentiation were identified from hapFLK scores, which indicate correlated haplotypes according to strains, using hapflk v1.4 [36]. As the computation was not feasible with the whole genome sequences, we randomly divided sequences in the genome assemblies into eight groups. F_ST_ distributions from these eight groups were highly similar (Additional file 18: Fig. S18). P-values showing the statistical significance of genetic differentiation were calculated from each position using scaling_chi2_hapflk.py in the same software package. Changes in population sizes were inferred using PSMC v0.65 [82]. We used the SLiM 2.0 [83] to perform forward simulation.

### Nuclear phylogenetic tree

We generated simulated fastq files from the reference genome of *S. litura *[84] using genReads v1 [85] with an error rate equal to 0.02. Then, the fastq files were mapped against the reference genome using bowtie2 v2.3.3 [66], followed by variant calling using samtools v1.5 mpileup [77]. The resulting VCF file was merged with the VCF file from the fall amryworm using vcftools v0.1.13 [79]. We discarded all positions unless genotypes are determined from all individuals from *S. frugiperda* and *S. litura*. After this filtering, 7,054,107 SNPs remained.

Euclidean distances between pairs of individuals were calculated from the difference in allele frequencies, while transversional changes are weighted to two. Then, bootstrapping distance matrixes were generated based on random samplings of SNPs with 1000 replications. Perl scripts used to generate the distance matrix are available at https://github.com/kiwoong-nam/VCFPhylo. A BIONJ phylogenetic tree was reconstructed from this new alignment using FastME v2.0 [86] with 1000 bootstrap replications. We generated a consensus tree from the resulting bootstrapping trees using consense in Phylip v3.697 pacakge [87]. A k-mer-based phylogenetic tree was generated from fastq files using AAF v20171001 [88].

### Mitochondrial phylogenetic tree

Reads at fastq were mapped against the mitochondrial genome (KM362176) using bowtie2 v2.3.3 [66], and variants were called using samtools v1.5 mpileup [77]. From the mitochondrial VCF file, a multiple sequence alignment was generated using custom perl scripts (available on request). Then, the whole mitochondrial genome from *S. litura* (KF701043) was added to this multiple sequence alignment, and a new alignment was generated using prank v140110 [89]. A phylogenetic tree was reconstructed from this new alignment using FastME v2.0 [86] with 1,000 bootstrapping replications.

## Supplementary information

**Additional file 1: Fig. S1.** Speciation models explaining whole genome differentiation. A. In the presence of very strong population-specific positive selection, a migration rate between two populations is effectively reduced, and whole genome differentiation may occur. B. Instead of a single event of very strong population-specific positive selection, multiple events of mild positive selection reduce the genomic rate of migration rate between populations, and whole genome differentiation may occur as well (genome hitchhiking model). C. If a very large number of loci are targeted by selective sweeps, almost entire genomic sequences are affected by at least one selective sweep. Then, whole genome differentiation may occur. The box represents a genomic region affected by a single selective sweep. D. If positively selected loci causing reproductive isolation are genetically linked within a genome, a long sequence containing these loci can be genetically differentiated. The grey box represents a locus with genetic differentiation. E. A sequence with chromosomal rearrangement (the yellow arrow) is genetically differentiated between populations because recombination is suppressed in this area due to the chromosomal rearrangement. If positive selection targets a locus that is genetically linked to the chromosomal rearrangement, a long DNA sequence containing chromosomal rearrangement and selectively targeted locus can be differentiated.

**Additional file 2: Fig. S2.** The read death (upper) and the alignment rate (lower) of the mappings reads against the reference genome. As ‘R1’ individual has a particularly lower read depth, we excluded this individual in this paper. R1 has the lowest alignment rate, as well.

**Additional file 3: Fig. S3.** The expected reduction in π when a beneficial mutation (cM = 0) is fixed in a population, (A) with biologically realistic selection coefficients (0 < s ≤ 1), and (B) biologically unrealistic selection coefficients (5 ≤ s ≤ 100).

**Additional file 4: Fig. S4.** The relationship of exon density with π (A) and FST (B) from the scaffolds without outliers.

**Additional file 5: Fig. S5.** The distribution of hapFLK scores along the chromosomes with ten longest outliers, indicated by red asterisks. Log-scaled p values show the statistical significance of a position having a higher hapFLK score than the genomic average.

**Additional file 6: Fig. S6.** Log-transformed p-values and hapFLK scores of the outliers that have higher dXY than the genomic average. The red bars indicate the borders of the outliers, and dotted lines show a p-value equal to 0.001.

**Additional file 7: Fig. S7.** The distribution of π of corn strain (sfC) and rice strain (sfR) from the outliers and 100kb windows from the whole genome sequences.

**Additional file 8: Fig. S8.** The distribution of dXY from outliers (red) and 10kb windows from whole genome sequences (blue).

**Additional file 9: Fig. S9.** k-mer based phylogenetic tree reconstructed from raw fastq files. Red, blue, and yellow bars indicate sfC, sfR, and S. litura, respectively.

**Additional file 10: Fig. S10.** The analysis of the ancestry coefficient. a). The relationship between K and cross-entropy. b). The ancestry coefficient when K = 2.

**Additional file 11: Fig. S11.** Principal component analysis from mitochondrial genomes

**Additional file 12: Fig. S12.** Mitochondrial phylogenetic tree. The mitochondrial sequence of each individual was inferred by mapping against mitochondrial genomes (NCBI accession number: KM362176) and by variant calling. Then, multiple sequence alignment was generated together with Spodoptera litura (NCBI accession number: KF701043) using prank software. The phylogenetic tree was reconstructed with 1,000 bootstrapping replications using the FastME software.

**Additional file 13: Fig. S13.** The analysis of the ancestry coefficient from the mitochondrial genome. A. The relationship between K and cross-entropy. B. The ancestry coefficient when K = 2.

**Additional file 14: Fig. S14.** We performed a simple forward simulation using slim software with a wide range of migration rates to test mitochondrial divergence time can explain the level of observed nuclear genetic differentiation (F_ST_ = 0.0174). The simulation was performed during 5 × Ne generations in 100kb sequences. Assuming that the generation time for each generation is 0.1 years (lab condition) and that Ne is 4 million, 5 × Ne generation time corresponds to 5 × 4 × 10^6^ × 0.1 = 2 × 10^6^ years, which is the reported mitochondrial divergence time based on the molecular clock[45]. The mutation rate, recombination rate, Ne of the ancestral population, Ne of two derived populations after the split from the ancestral population, and the generation time after the split are 1.16 × 10^−4^, 1.188×10^−3^, 200, 100, and 500, respectively. For each migration rate, 500 independent simulations were performed, and the calculated F_ST_ was averaged. The red horizontal bar indicates the genomic average FST, which is 0.0174. Please note that the used parameters were rescaled by 4,000 folds from 2.9 × 10^−9^ and 2.97 × 10^−8^, for mutation rate and recombination rate, respectively. And the used parameters were rescaled by 1/4000 folds from 8 × 10^6^, 4×10^6^, and 2×10^7^, for Ne of the ancestral population, and Ne of two derived population after the split from the ancestral population, and the number of generations after the split, respectively.

**Additional file 15: Fig. S15.** The read-depth of mappings against FR (NCBI ID: FR.X78688.1), which are expected to be present only in the sfR.

**Additional file 16: Fig. S16.** A possible explanation for the discrepancy of identified strains between the mitochondrial genome and nuclear TPI gene. Names of the leaves of the trees show the strains identified from the mitochondrial genome. For example, sfC.1 and sfC.2 are the individuals identified as sfC, according to mitochondrial markers. As noted in the main text of the paper, mitochondrial divergence time is older than the averaged nuclear divergence time. The divergence time of TPI gene is lower than the averaged nuclear divergence time. The dashed horizontal bars indicate the average nuclear differentiation time. (upper) If a selective sweep on TPI gene occurs at the common ancestor of sfR.2 and sfR.3, then sfC.1, sfC.2, and sfR.1 share the common genotypes. (lower) If the selective sweep occurs at the common ancestor of sfC.1, sfC.2, and sfR.1, these three individuals share the common genotypes. In these two cases, sfR.1 will be identified as sfC when TPI gene is used as a marker.

**Additional file 17: Fig. S17.** Historical changes in effective population sizes. We used the Pairwise Sequentially Markovian Coalescent (PSMC) model to infer changes in effective population sizes from each individual. The red and blue lines indicate individuals from sfC and sfR, respectively.

**Additional file 18: Fig. S18.** Distribution of F_ST_ calculated from each of eight groups from which hapFLK scores were calculated. These groups were generated by a random grouping of scaffolds into eights.

**Additional file 19: Table S1.** The result of BUSCO analysis to evaluate the correctness of gene annotations from sfCand sfR genome assemblies generated in this study and our previous study (Gouin et al. [31]). **Table S3.** The number of genes within outliers of genetic differentiation that are potentiallyassociated with interactions with host-plants. **Table S4.** Nucleotide diversity (π) calculated from whole genome sequences in insects. The species ) calculated from whole genome sequences in insects. The specieswere sorted according to π) calculated from whole genome sequences in insects. The species . * denotes π) calculated from whole genome sequences in insects. The species calculated from the sum of π) calculated from whole genome sequences in insects. The species divided by the assembly sizein the fall armyworm. ** denotes π) calculated from whole genome sequences in insects. The species calculated from four-fold degenerative sites.

**Additional file 20: Table S2.** Genes within genetic outliers of differentiation identified from the mapping against sfR reference genome.

## Data Availability

The reference genome and gene annotation are available from the BioInformatics Platform for Agroecosystem Arthropods together with the genome browser (www.bipaa.genouest.org/sp/spodoptera_frugiperda) and NCBI (JACWZX000000000). The resequencing data is available at NCBI SRA (project id: PRJEB29161). Resequencing data is available from NCBI Sequence Read Archive, and the corresponding project ID is PRJNA494340.

## Abbreviations

BIONJ: Bio neighbor-joining
BUSCO: Benchmarking Universal Single-Copy Orthologs
cM: Centimorgan
CO1: Cytochrome c oxidase subunit I
DNA: Deoxyribonucleic acid
FDR: False discovery rate
GD: Genomic differentiation
NPRL2: Nitrogen permease regulator 2-like
PSMC: Pairwise Sequentially Markovian Coalescent
*s*: Selection coefficient
sfC: Corn strain in *Spodoptera frugiperda*
sfR: Rice strain in *Spodoptera frugiperda*
SNP: Single nucleotide polymorphism
TOR pathway: Target of rapamycin pathway
TORC1: Target of rapamycin complex 1
TPI: Triosephosphate isomerase
VCF: Variant calling format
