# Supplementary figures and images for: Positive selection alone is sufficient for whole genome differentiation at the early stage of speciation process in the fall armyworm
Source: BMC Evol Biol. 2020 Nov 13;20:152. doi: 10.1186/s12862-020-01715-3 (PMC7663868; doi:10.1186/s12862-020-01715-3)

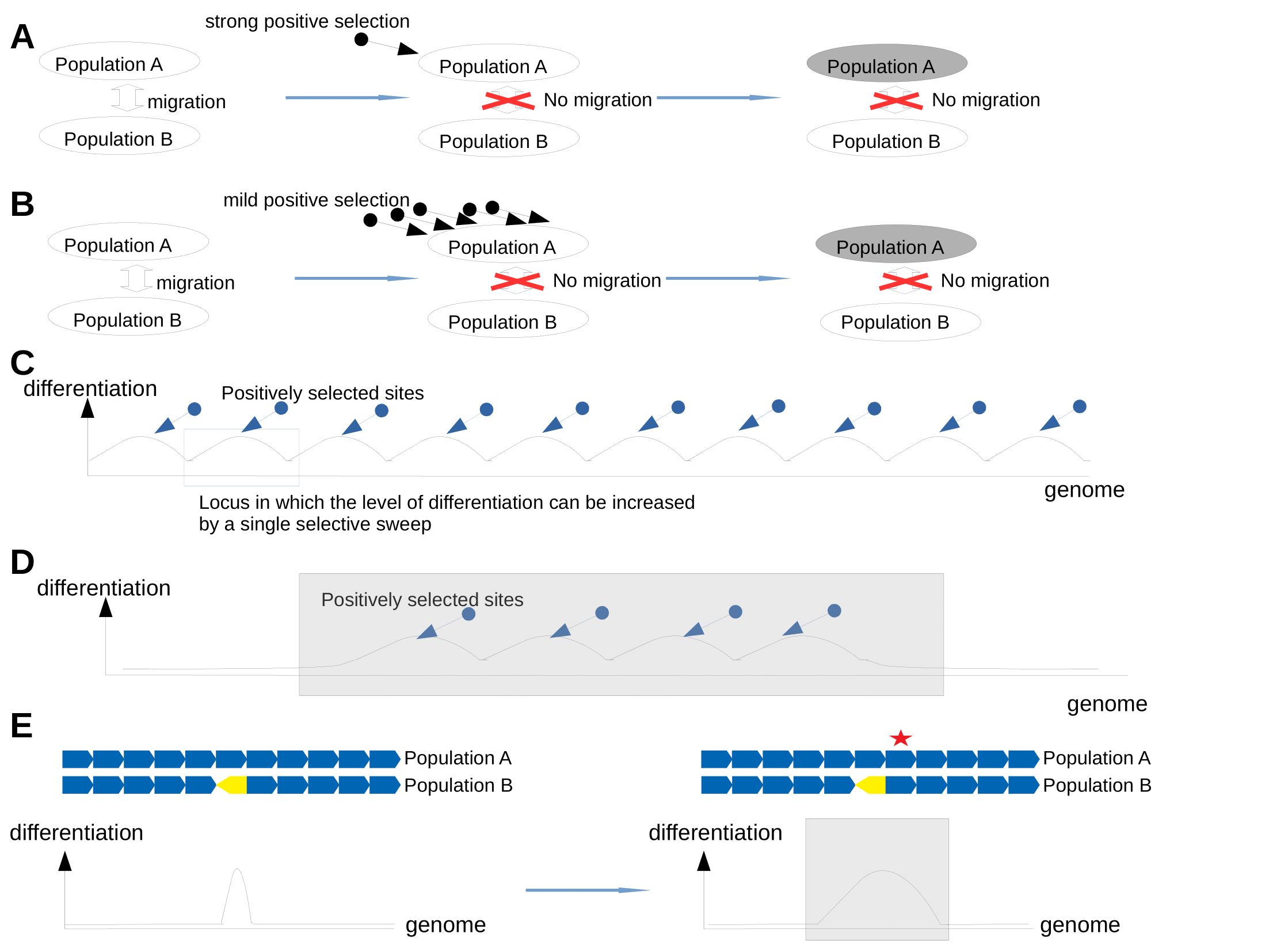

Supplement: Supplementary file 1 — Additional file 1: Fig. S1. Speciation models explaining whole genome differentiation. A. In the presence of very strong population-specific positive selection, a migration rate between two populations is effectively reduced, and whole genome differentiation may occur. B. Instead of a single event of very strong population-specific positive selection, multiple events of mild positive selection reduce the genomic rate of migration rate between populations, and whole genome differentiation may occur as well (genome hitchhiking model). C. If a very large number of loci are targeted by selective sweeps, almost entire genomic sequences are affected by at least one selective sweep. Then, whole genome differentiation may occur. The box represents a genomic region affected by a single selective sweep. D. If positively selected loci causing reproductive isolation are genetically linked within a genome, a long sequence containing these loci can be genetically differentiated. The grey box represents a locus with genetic differentiation. E. A sequence with chromosomal rearrangement (the yellow arrow) is genetically differentiated between populations because recombination is suppressed in this area due to the chromosomal rearrangement. If positive selection targets a locus that is genetically linked to the chromosomal rearrangement, a long DNA sequence containing chromosomal rearrangement and selectively targeted locus can be differentiated. [file 12862_2020_1715_MOESM1_ESM.png]

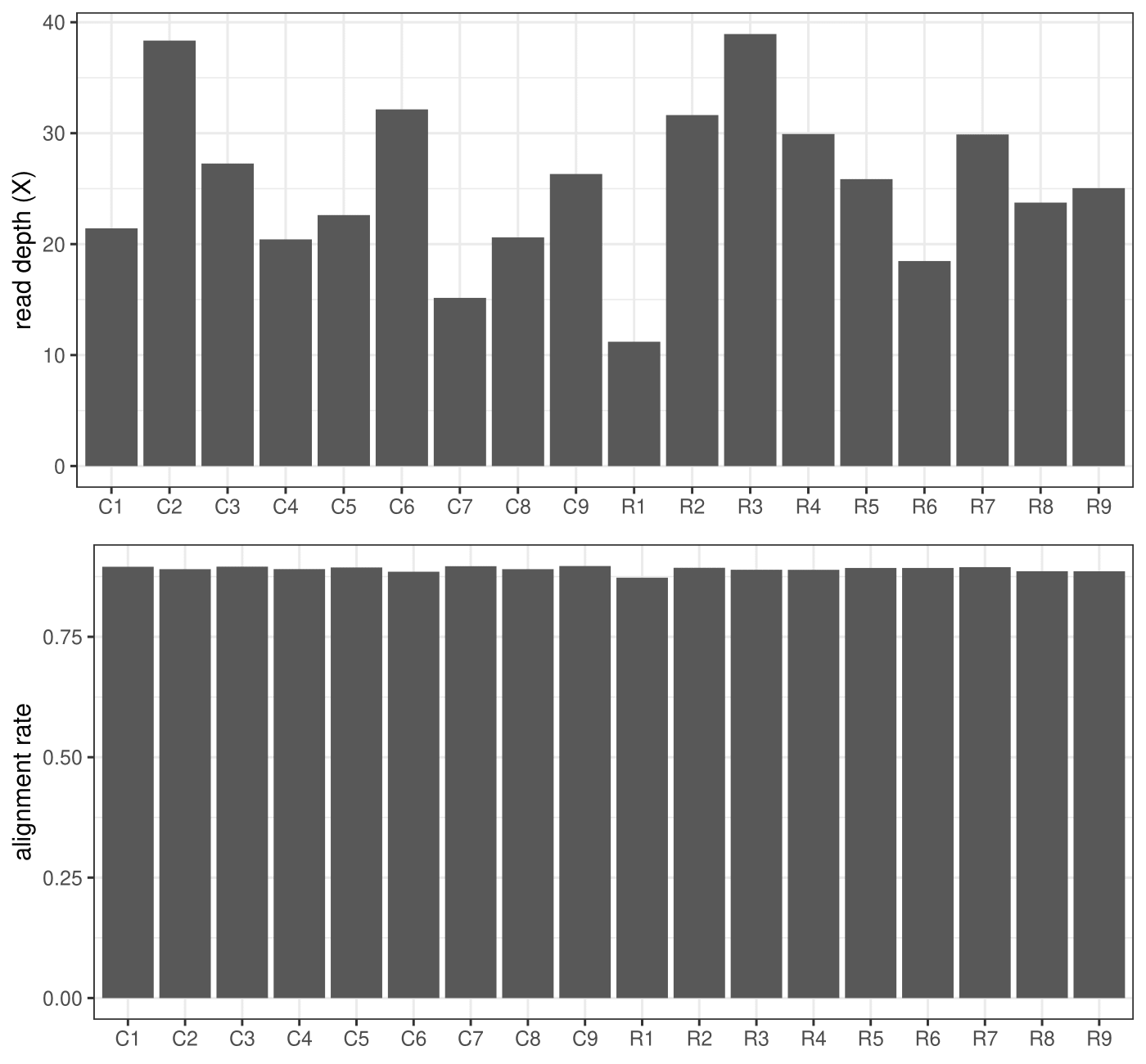

Supplement: Supplementary file 2 — Additional file 2: Fig. S2. The read death (upper) and the alignment rate (lower) of the mappings reads against the reference genome. As ‘R1’ individual has a particularly lower read depth, we excluded this individual in this paper. R1 has the lowest alignment rate, as well. [file 12862_2020_1715_MOESM2_ESM.png]

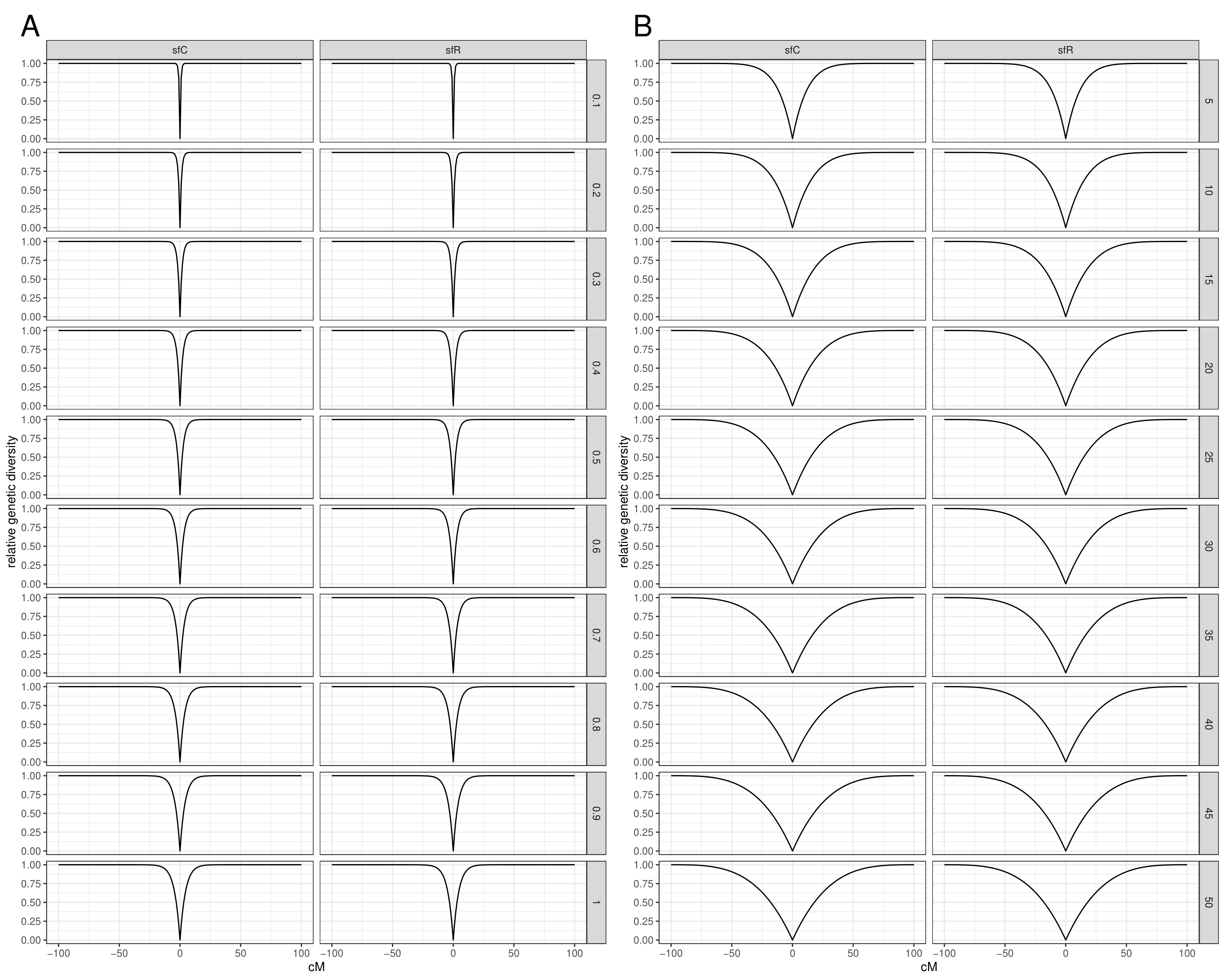

Supplement: Supplementary file 3 — Additional file 3: Fig. S3. The expected reduction in π when a beneficial mutation (cM = 0) is fixed in a population, (A) with biologically realistic selection coefficients (0 < s ≤ 1), and (B) biologically unrealistic selection coefficients (5 ≤ s ≤ 100). [file 12862_2020_1715_MOESM3_ESM.png]

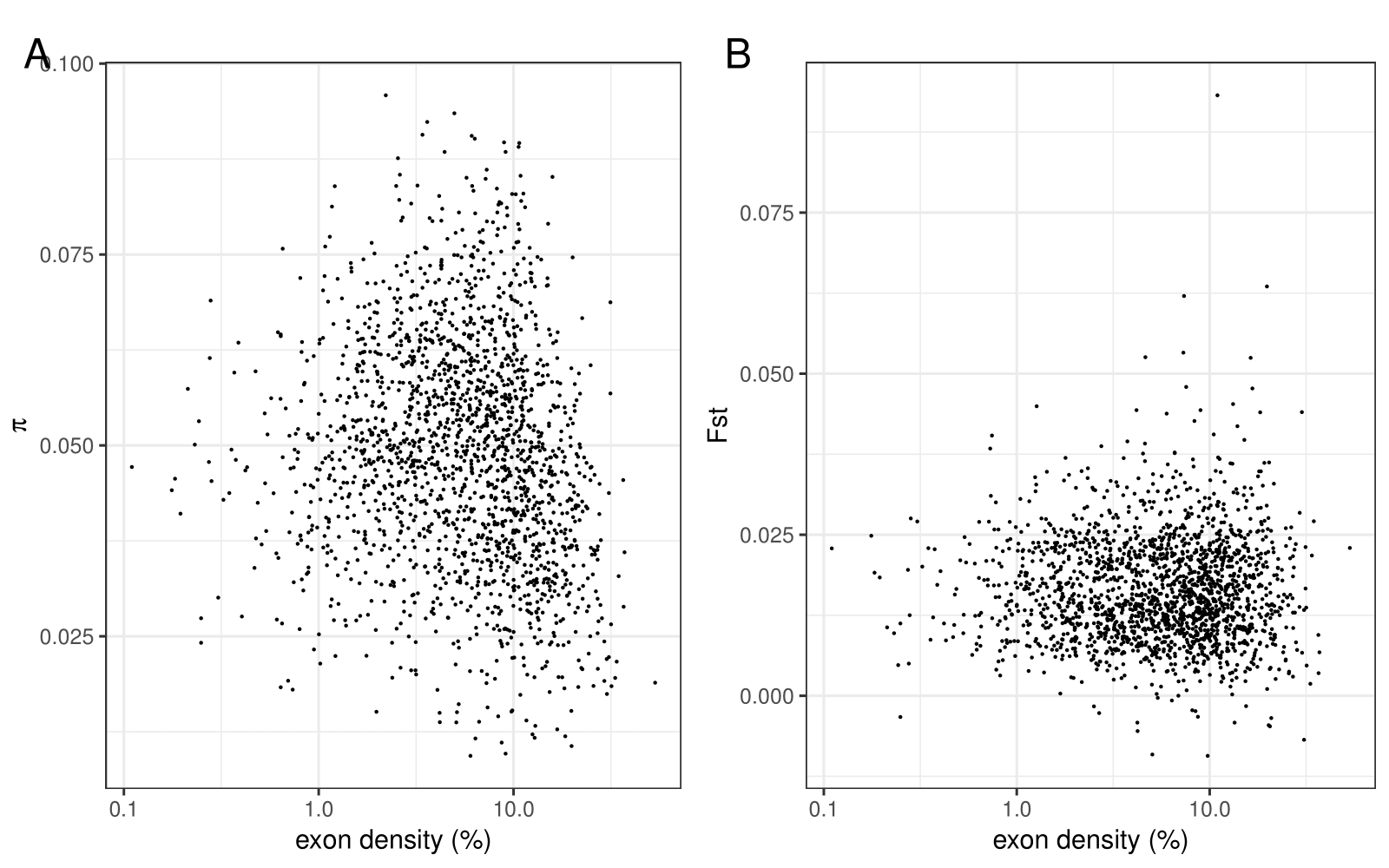

Supplement: Supplementary file 4 — Additional file 4: Fig. S4. The relationship of exon density with π (A) and FST (B) from the scaffolds without outliers. [file 12862_2020_1715_MOESM4_ESM.png]

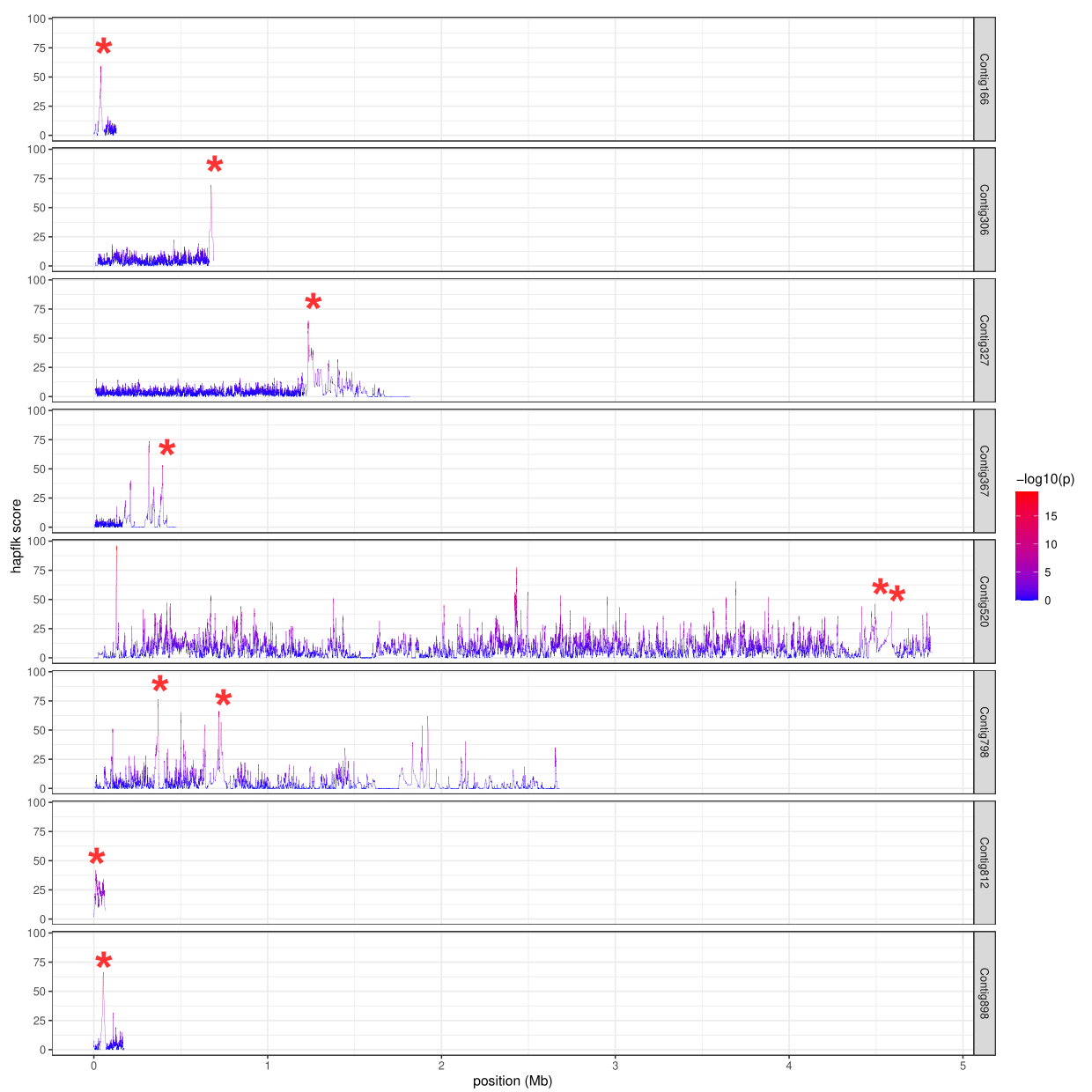

Supplement: Supplementary file 5 — Additional file 5: Fig. S5. The distribution of hapFLK scores along the chromosomes with ten longest outliers, indicated by red asterisks. Log-scaled p values show the statistical significance of a position having a higher hapFLK score than the genomic average. [file 12862_2020_1715_MOESM5_ESM.png]

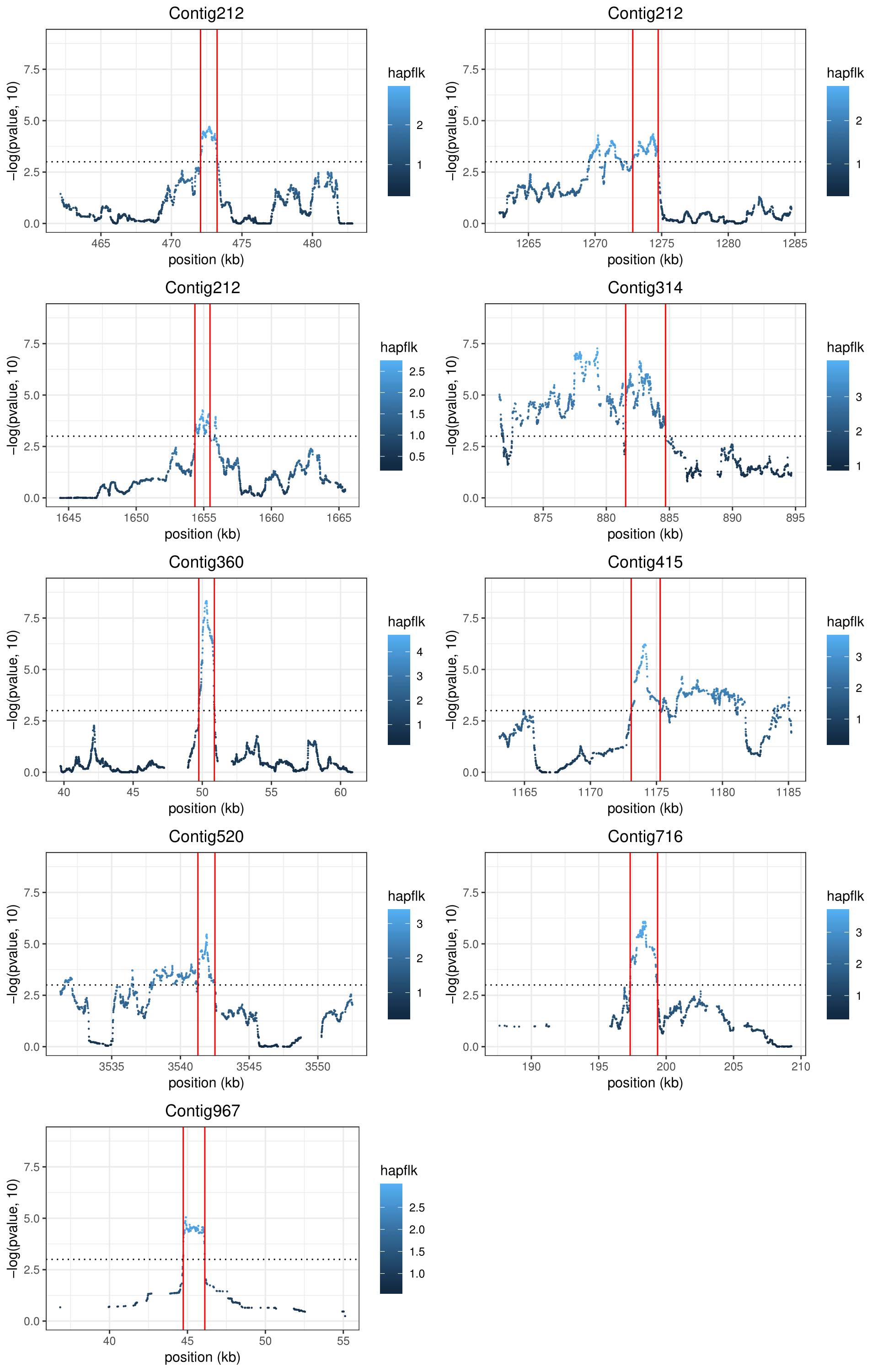

Supplement: Supplementary file 6 — Additional file 6: Fig. S6. Log-transformed p-values and hapFLK scores of the outliers that have higher dXY than the genomic average. The red bars indicate the borders of the outliers, and dotted lines show a p-value equal to 0.001. [file 12862_2020_1715_MOESM6_ESM.png]

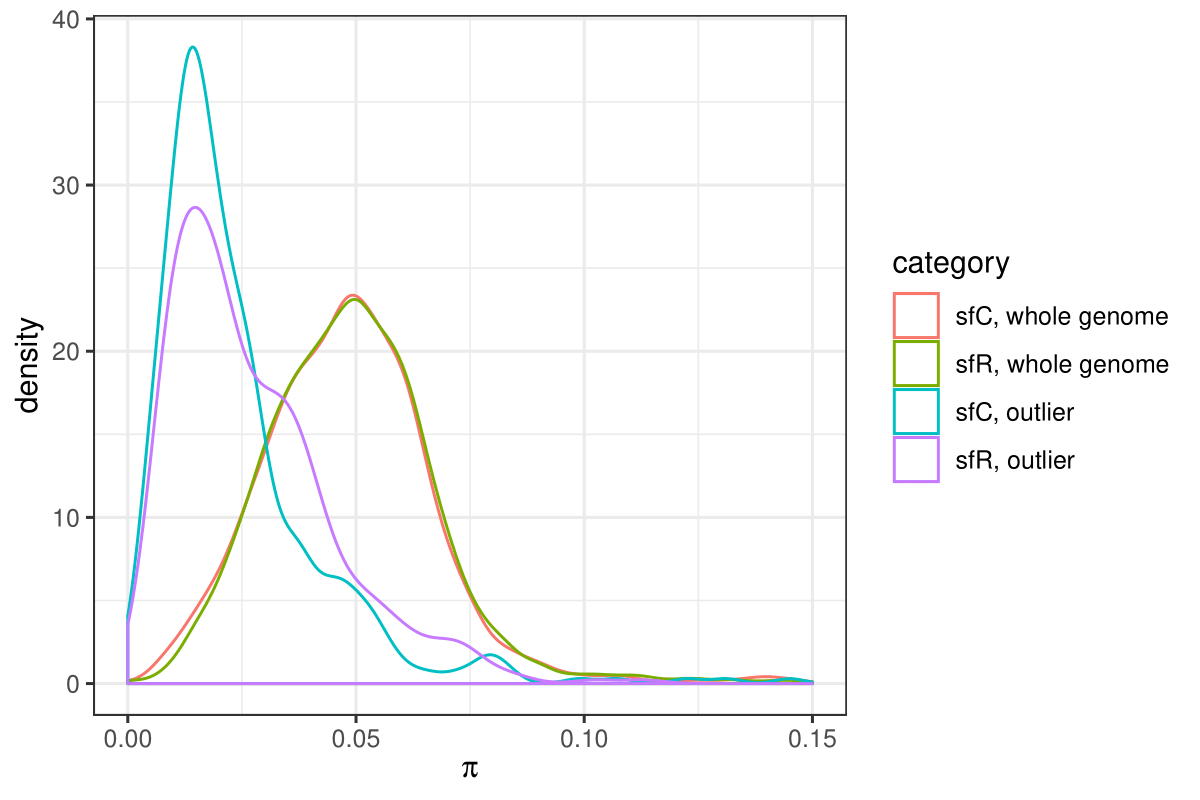

Supplement: Supplementary file 7 — Additional file 7: Fig. S7. The distribution of π of corn strain (sfC) and rice strain (sfR) from the outliers and 100kb windows from the whole genome sequences. [file 12862_2020_1715_MOESM7_ESM.png]

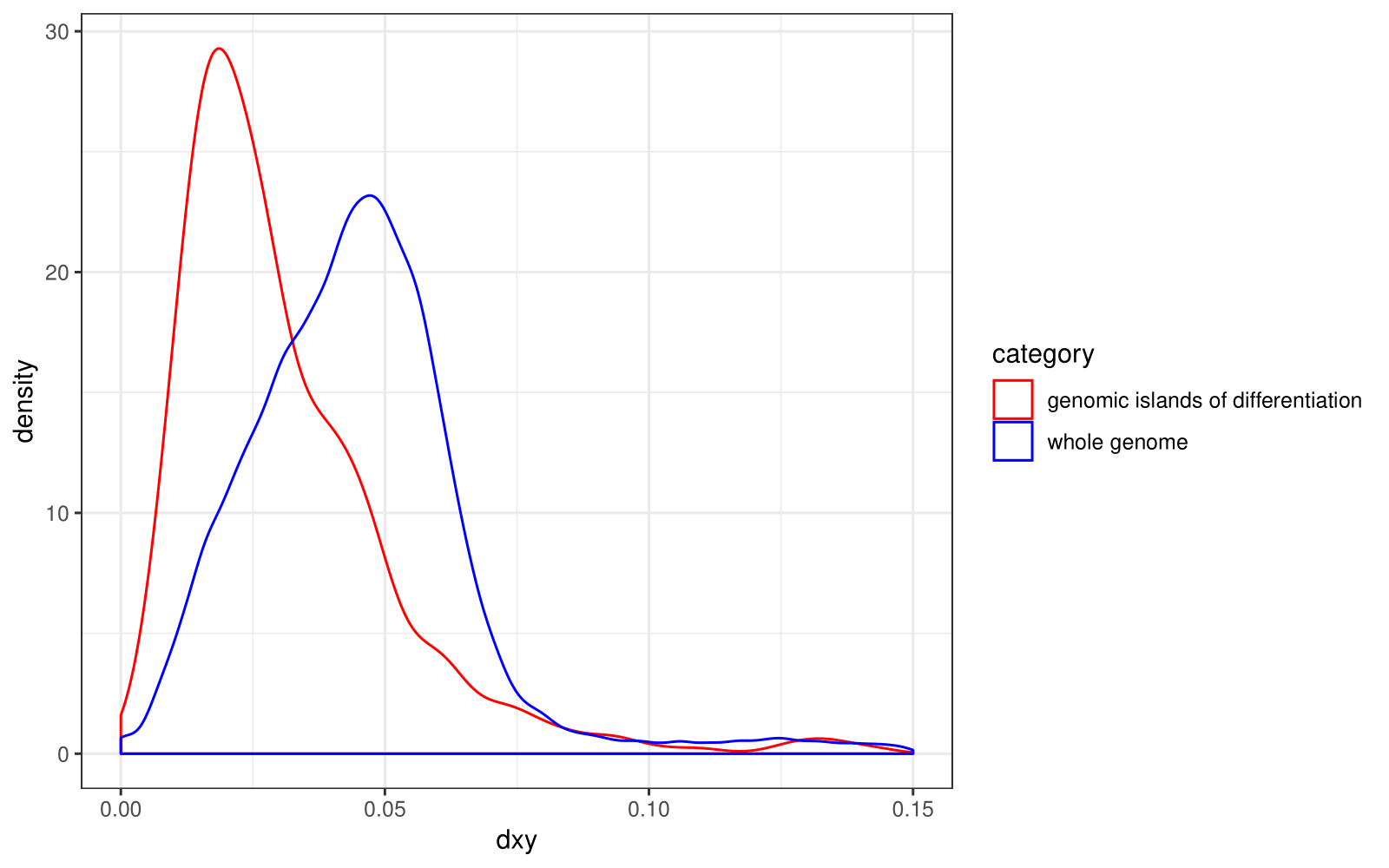

Supplement: Supplementary file 8 — Additional file 8: Fig. S8. The distribution of dXY from outliers (red) and 10kb windows from whole genome sequences (blue). [file 12862_2020_1715_MOESM8_ESM.png]

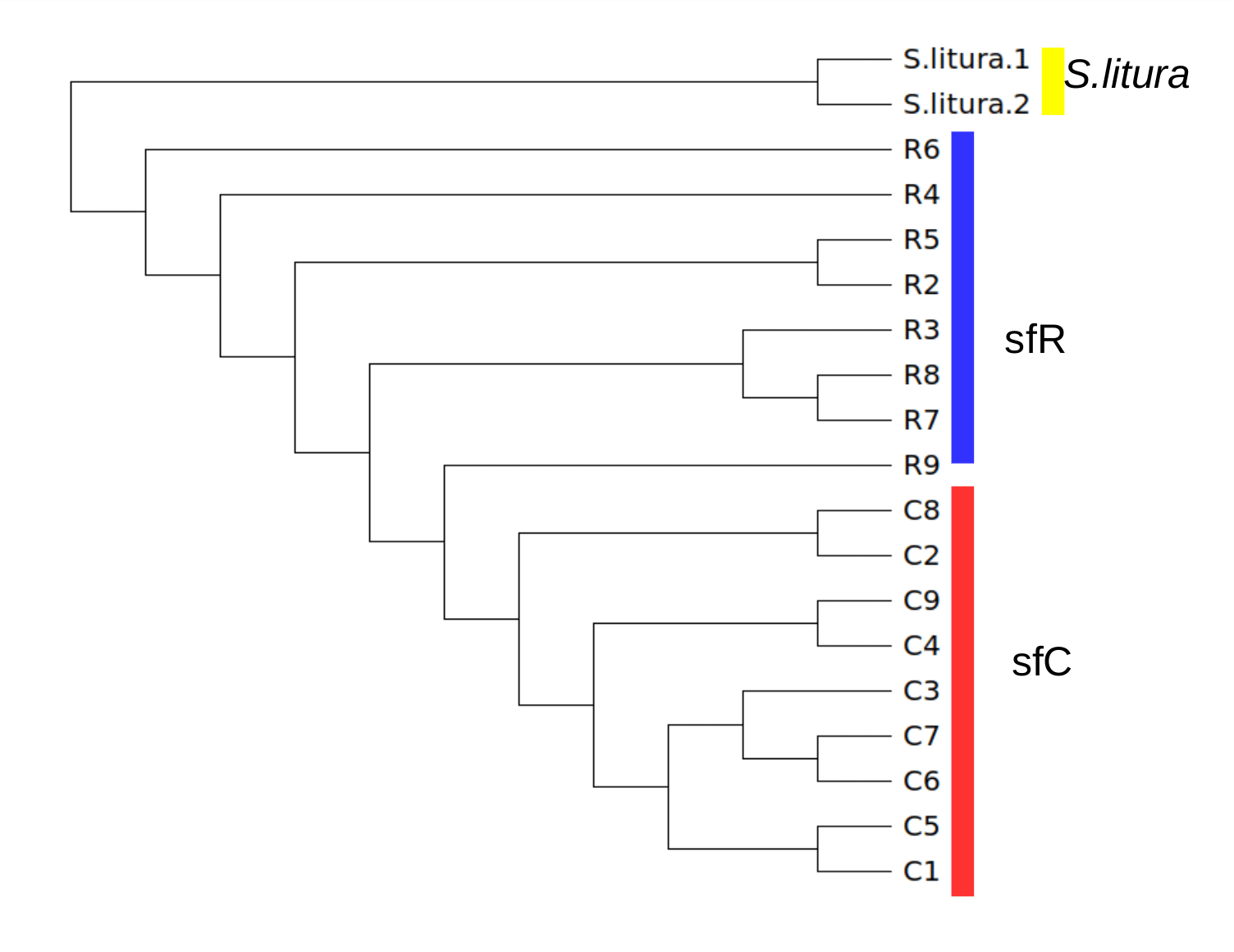

Supplement: Supplementary file 9 — Additional file 9: Fig. S9. k-mer based phylogenetic tree reconstructed from raw fastq files. Red, blue, and yellow bars indicate sfC, sfR, and S. litura, respectively. [file 12862_2020_1715_MOESM9_ESM.png]

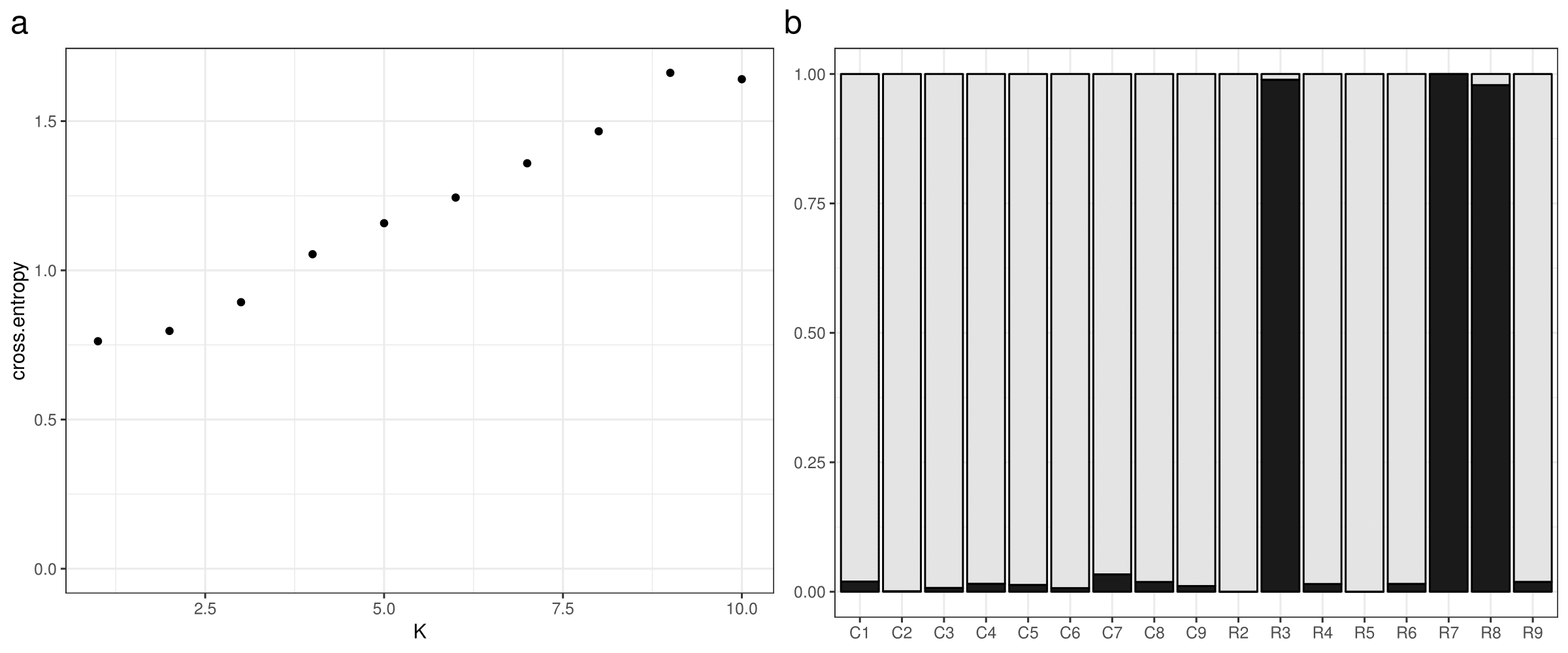

Supplement: Supplementary file 10 — Additional file 10: Fig. S10. The analysis of the ancestry coefficient. a). The relationship between K and cross-entropy. b). The ancestry coefficient when K = 2. [file 12862_2020_1715_MOESM10_ESM.png]

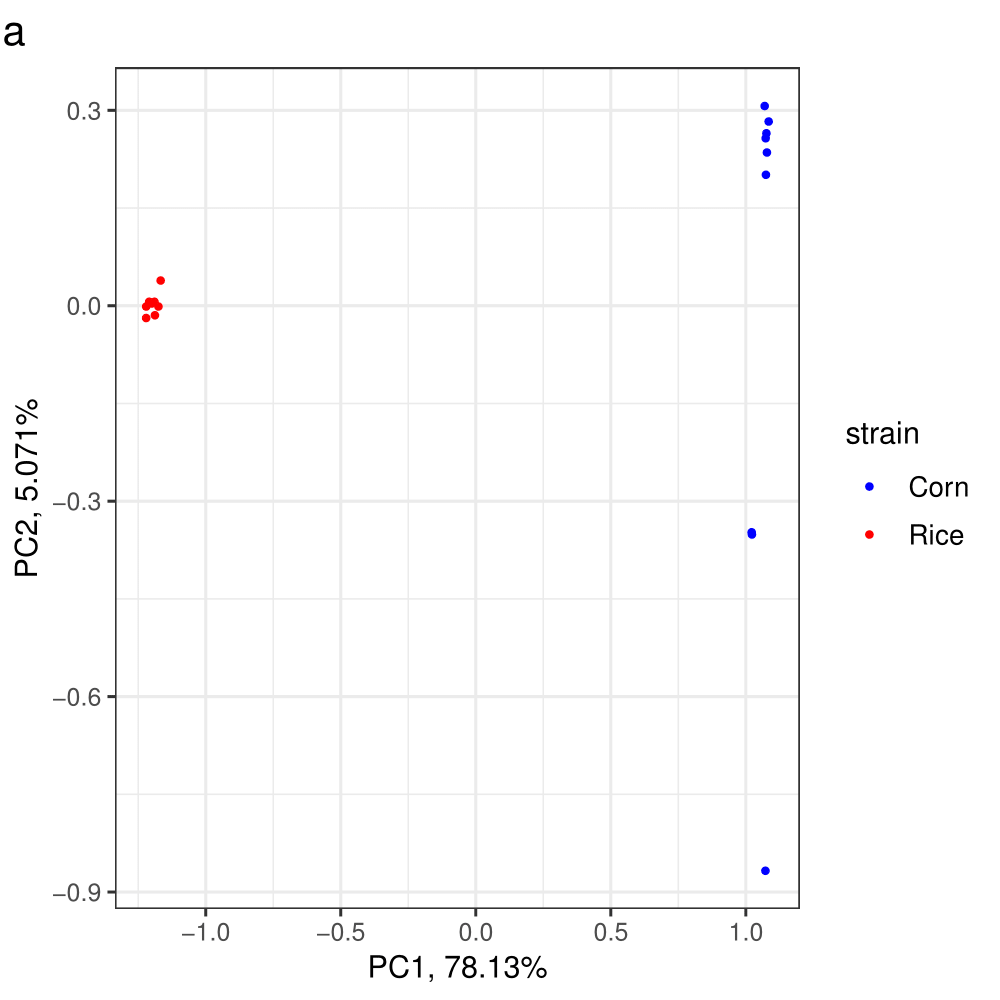

Supplement: Supplementary file 11 — Additional file 11: Fig. S11. Principal component analysis from mitochondrial genomes [file 12862_2020_1715_MOESM11_ESM.png]

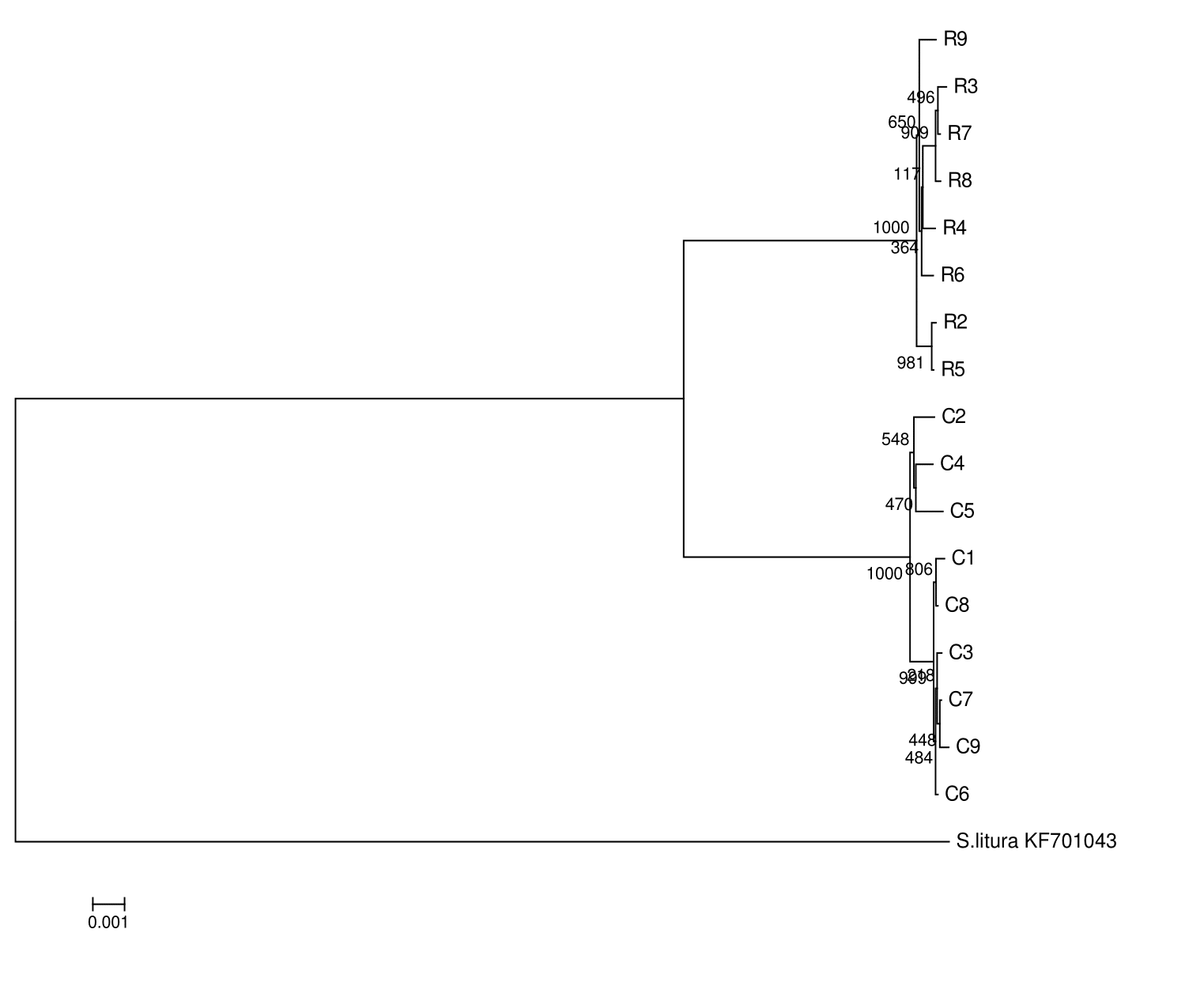

Supplement: Supplementary file 12 — Additional file 12: Fig. S12. Mitochondrial phylogenetic tree. The mitochondrial sequence of each individual was inferred by mapping against mitochondrial genomes (NCBI accession number: KM362176) and by variant calling. Then, multiple sequence alignment was generated together with Spodoptera litura (NCBI accession number: KF701043) using prank software. The phylogenetic tree was reconstructed with 1,000 bootstrapping replications using the FastME software. [file 12862_2020_1715_MOESM12_ESM.png]

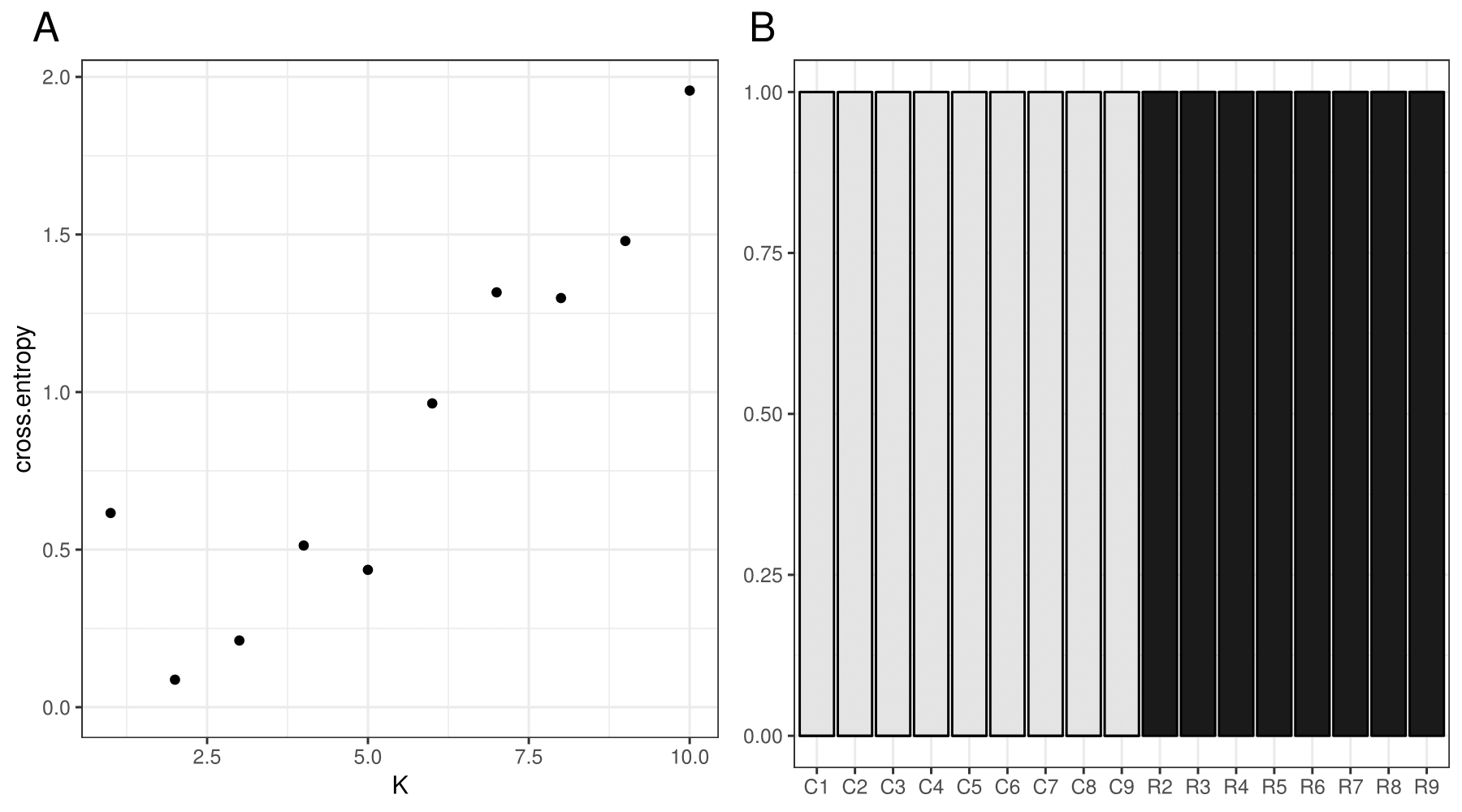

Supplement: Supplementary file 13 — Additional file 13: Fig. S13. The analysis of the ancestry coefficient from the mitochondrial genome. A. The relationship between K and cross-entropy. B. The ancestry coefficient when K = 2. [file 12862_2020_1715_MOESM13_ESM.png]

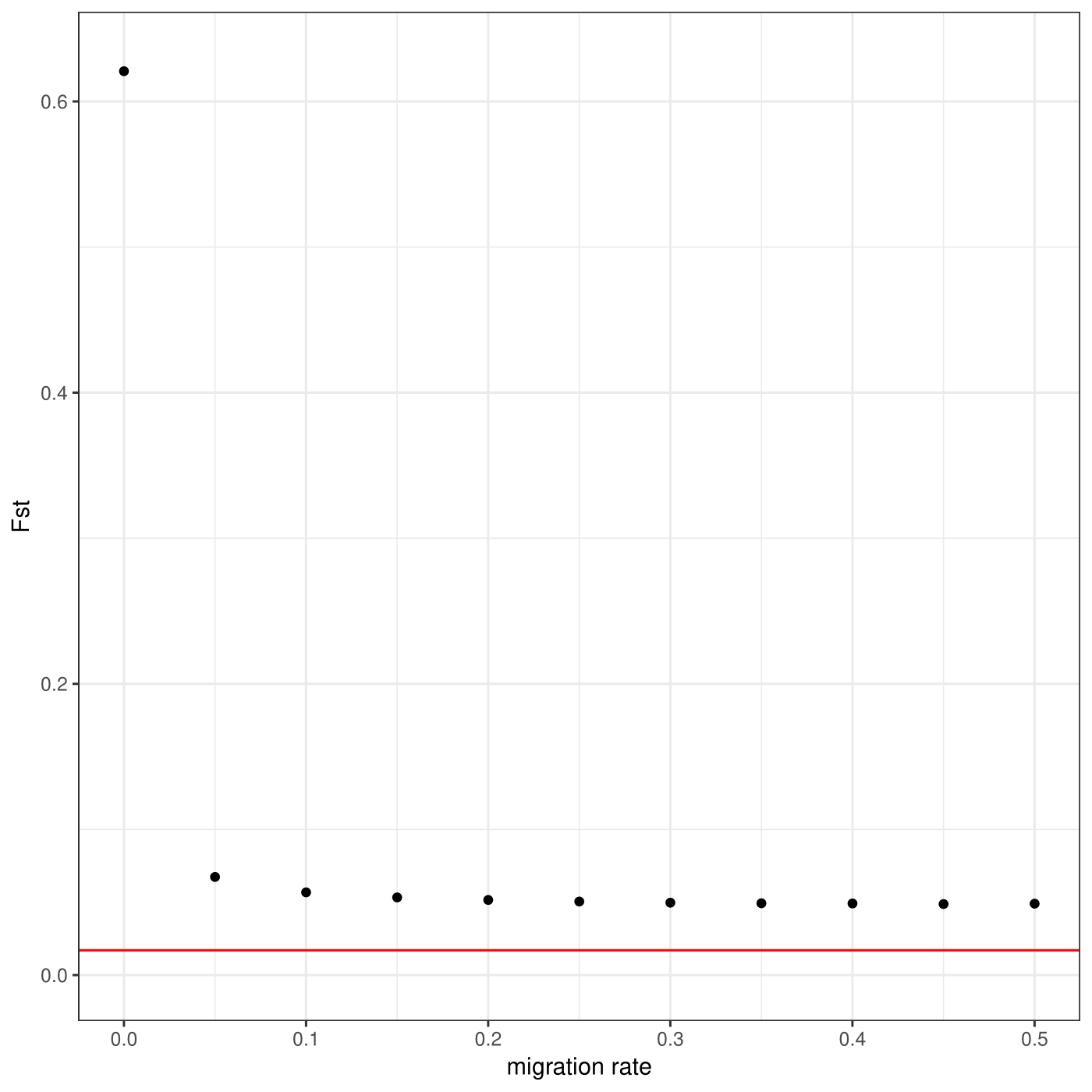

Supplement: Supplementary file 14 — Additional file 14: Fig. S14. We performed a simple forward simulation using slim software with a wide range of migration rates to test mitochondrial divergence time can explain the level of observed nuclear genetic differentiation (FST = 0.0174). The simulation was performed during 5 × Ne generations in 100kb sequences. Assuming that the generation time for each generation is 0.1 years (lab condition) and that Ne is 4 million, 5 × Ne generation time corresponds to 5 × 4 × 106 × 0.1 = 2 × 106 years, which is the reported mitochondrial divergence time based on the molecular clock[45]. The mutation rate, recombination rate, Ne of the ancestral population, Ne of two derived populations after the split from the ancestral population, and the generation time after the split are 1.16 × 10^−4^, 1.188×10^−3^, 200, 100, and 500, respectively. For each migration rate, 500 independent simulations were performed, and the calculated FST was averaged. The red horizontal bar indicates the genomic average FST, which is 0.0174. Please note that the used parameters were rescaled by 4,000 folds from 2.9 × 10^−9^ and 2.97 × 10^−8^, for mutation rate and recombination rate, respectively. And the used parameters were rescaled by 1/4000 folds from 8 × 10^6^, 4×10^6^, and 2×10^7^, for Ne of the ancestral population, and Ne of two derived population after the split from the ancestral population, and the number of generations after the split, respectively. [file 12862_2020_1715_MOESM14_ESM.png]

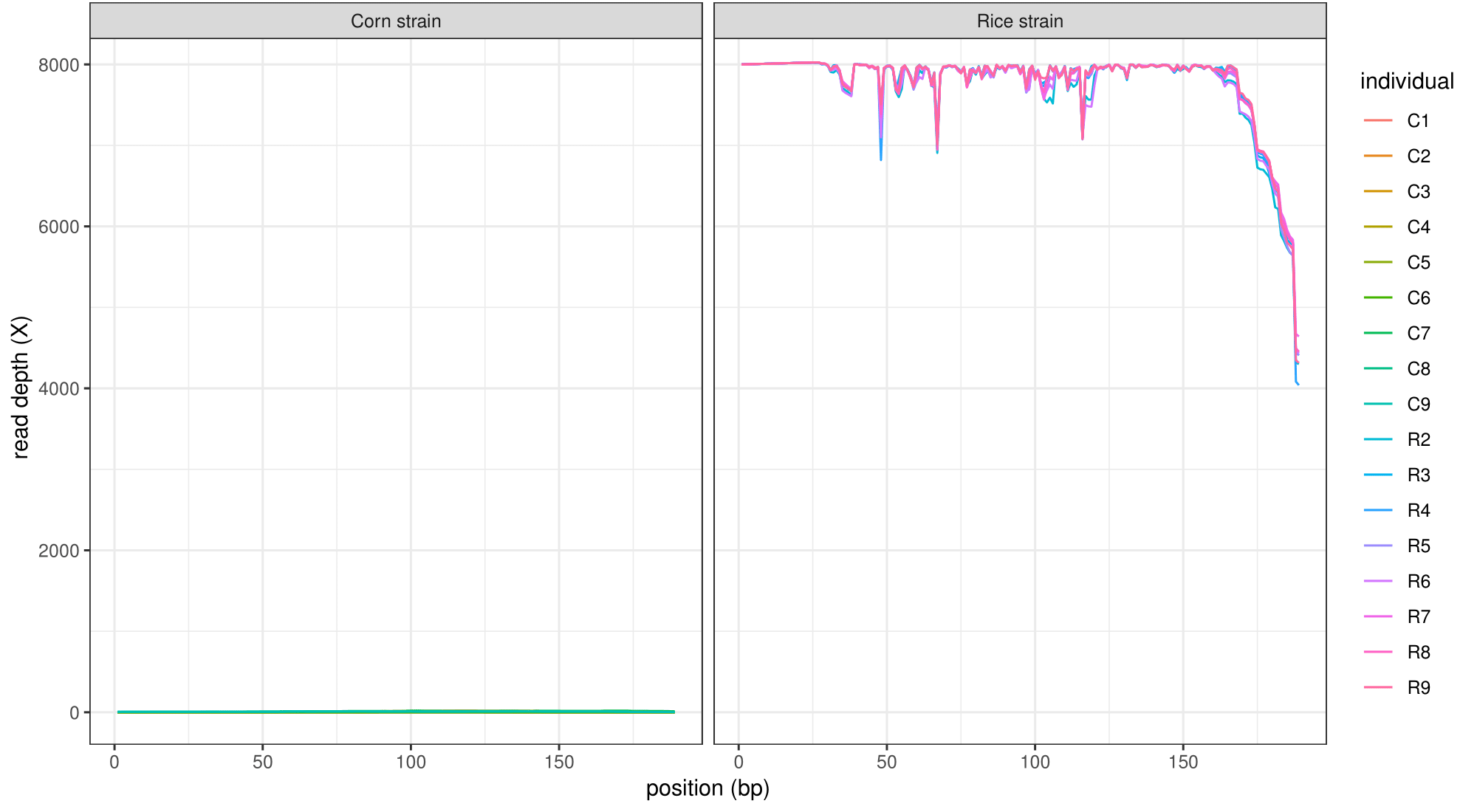

Supplement: Supplementary file 15 — Additional file 15: Fig. S15. The read-depth of mappings against FR (NCBI ID: FR.X78688.1), which are expected to be present only in the sfR. [file 12862_2020_1715_MOESM15_ESM.png]

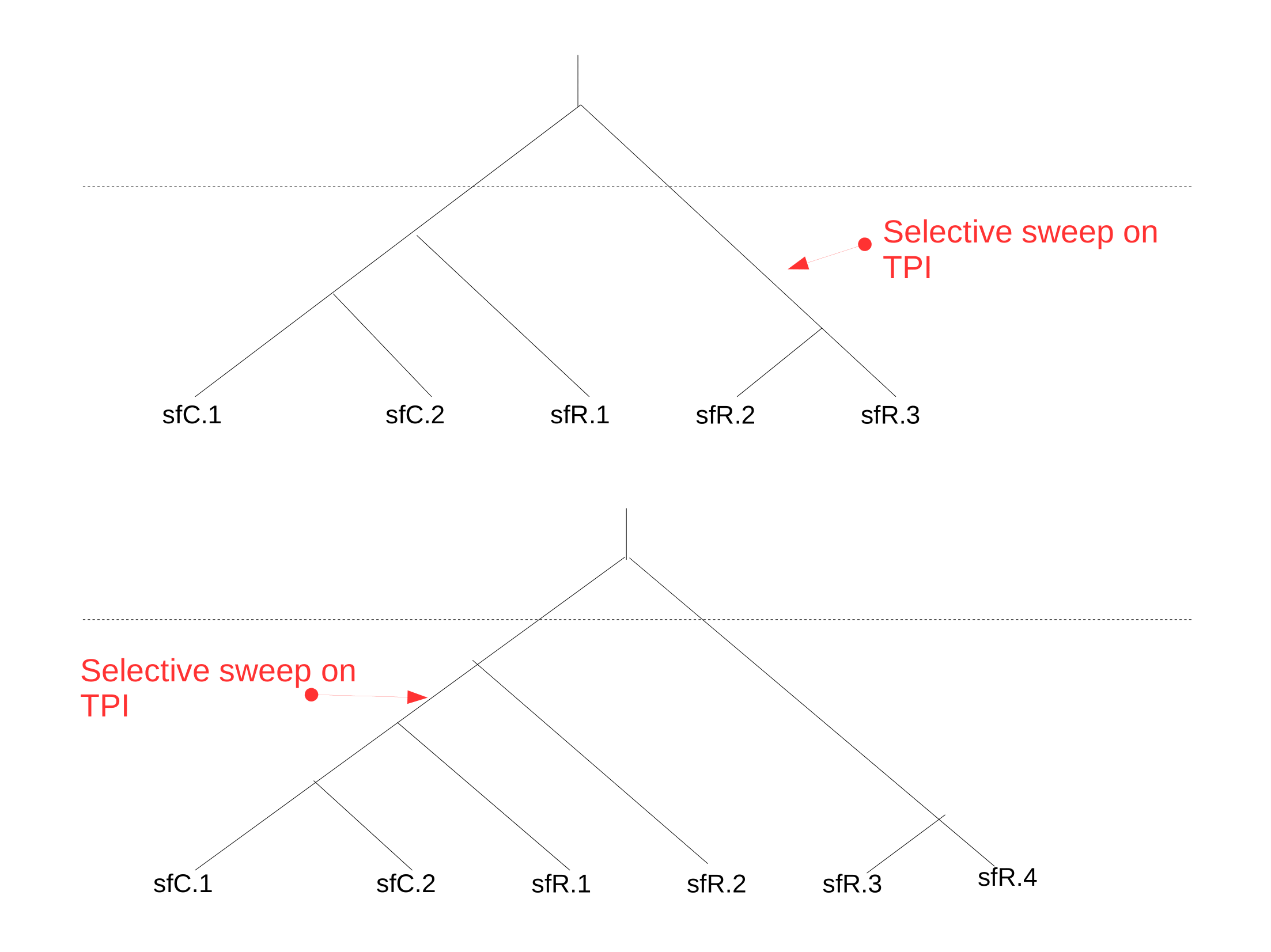

Supplement: Supplementary file 16 — Additional file 16: Fig. S16. A possible explanation for the discrepancy of identified strains between the mitochondrial genome and nuclear TPI gene. Names of the leaves of the trees show the strains identified from the mitochondrial genome. For example, sfC.1 and sfC.2 are the individuals identified as sfC, according to mitochondrial markers. As noted in the main text of the paper, mitochondrial divergence time is older than the averaged nuclear divergence time. The divergence time of TPI gene is lower than the averaged nuclear divergence time. The dashed horizontal bars indicate the average nuclear differentiation time. (upper) If a selective sweep on TPI gene occurs at the common ancestor of sfR.2 and sfR.3, then sfC.1, sfC.2, and sfR.1 share the common genotypes. (lower) If the selective sweep occurs at the common ancestor of sfC.1, sfC.2, and sfR.1, these three individuals share the common genotypes. In these two cases, sfR.1 will be identified as sfC when TPI gene is used as a marker. [file 12862_2020_1715_MOESM16_ESM.png]

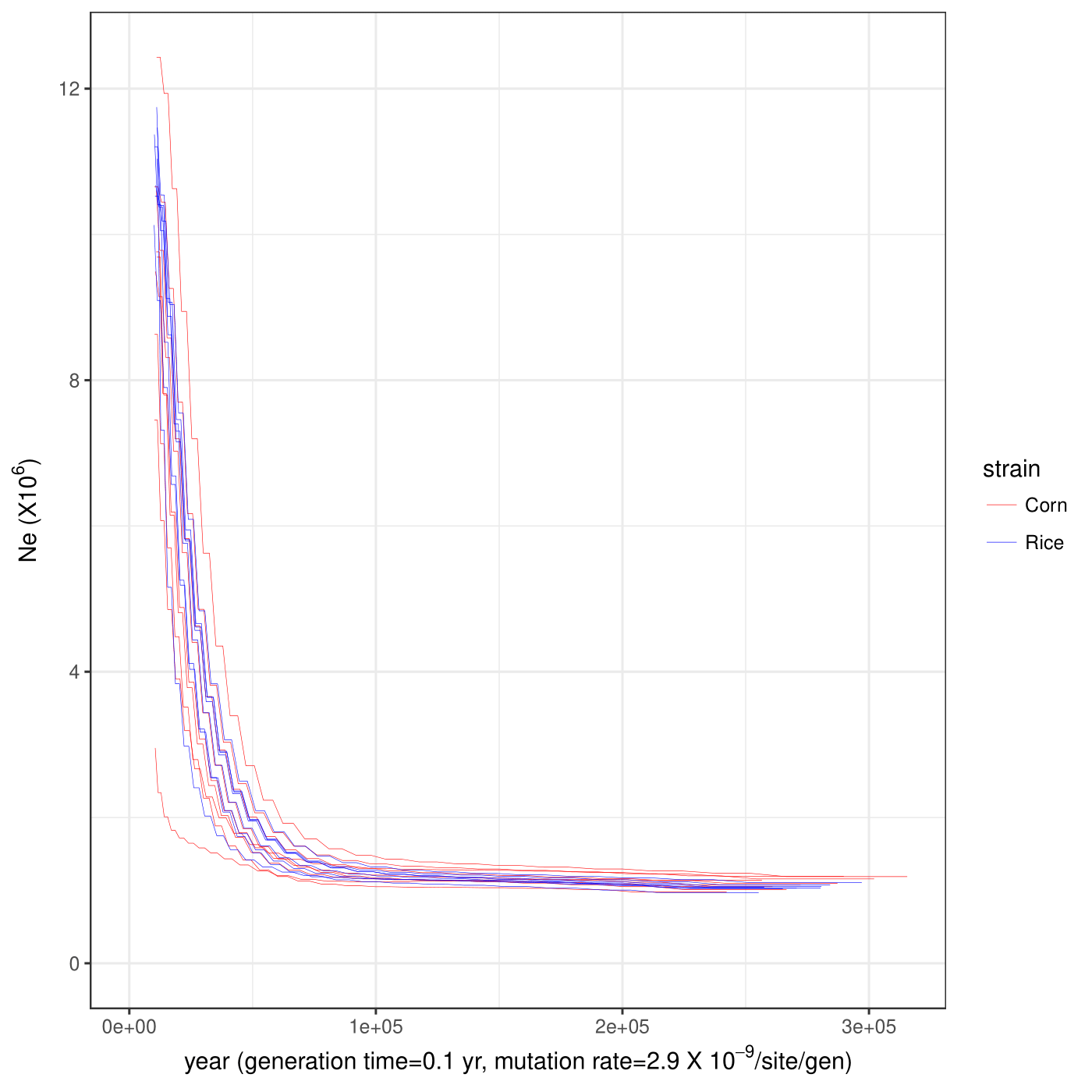

Supplement: Supplementary file 17 — Additional file 17: Fig. S17. Historical changes in effective population sizes. We used the Pairwise Sequentially Markovian Coalescent (PSMC) model to infer changes in effective population sizes from each individual. The red and blue lines indicate individuals from sfC and sfR, respectively. [file 12862_2020_1715_MOESM17_ESM.png]

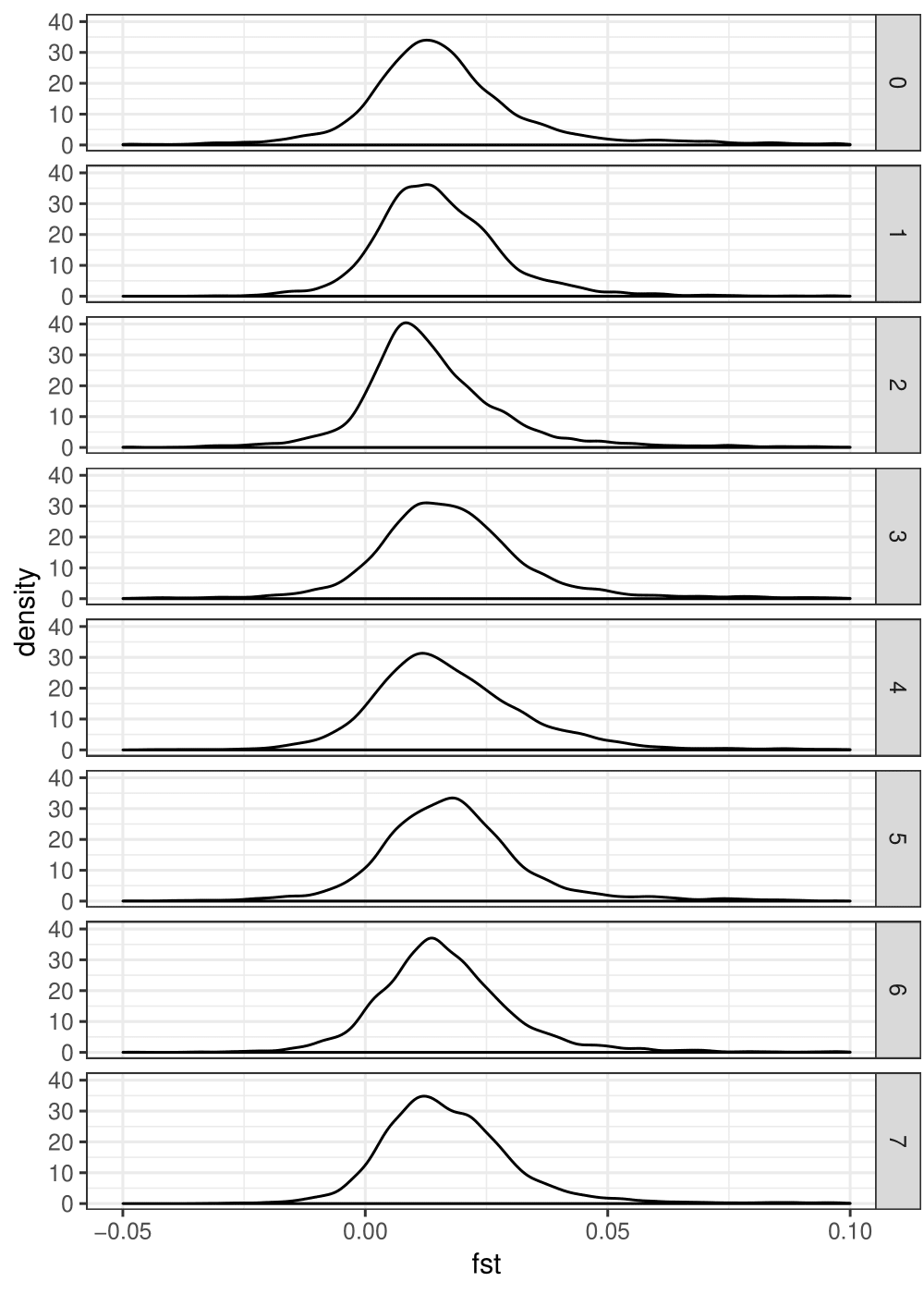

Supplement: Supplementary file 18 — Additional file 18: Fig. S18. Distribution of FST calculated from each of eight groups from which hapFLK scores were calculated. These groups were generated by a random grouping of scaffolds into eights. [file 12862_2020_1715_MOESM18_ESM.png]
