# Supplementary material for: Positive selection alone is sufficient for whole genome differentiation at the early stage of speciation process in the fall armyworm
Source: BMC Evol Biol. 2020 Nov 13;20:152. doi: 10.1186/s12862-020-01715-3 (PMC7663868; doi:10.1186/s12862-020-01715-3)
Supplement: Supplementary file 19 — Additional file 19: Table S1. The result of BUSCO analysis to evaluate the correctness of gene annotations from sfCand sfR genome assemblies generated in this study and our previous study (Gouin et al. [31]). Table S3. The number of genes within outliers of genetic differentiation that are potentiallyassociated with interactions with host-plants. Table S4. Nucleotide diversity (π) calculated from whole genome sequences in insects. The species ) calculated from whole genome sequences in insects. The specieswere sorted according to π) calculated from whole genome sequences in insects. The species . * denotes π) calculated from whole genome sequences in insects. The species calculated from the sum of π) calculated from whole genome sequences in insects. The species divided by the assembly sizein the fall armyworm. ** denotes π) calculated from whole genome sequences in insects. The species calculated from four-fold degenerative sites. [file 12862_2020_1715_MOESM19_ESM.pdf]

SUPPLEMENTARY TABLES

Table S1. The result of BUSCO analysis to evaluate the correctness of gene annotations from sfC and sfR genome assemblies generated in this study and our previous study (Gouin et al.[31]).

Number of BUSCO genes	Gouin et al		This study
	sfC	sfR	
Complete	1,434	1,551	1,562
Complete and single-copy	1,240	1,514	1,525
Complete and duplicated	194	37	37
Fragmented	164	67	35
Missing	60	40	61
Total	1,658	1,658	1,658

Table S3. The number of genes within outliers of genetic differentiation that are potentially associated with interactions with host-plants.

Functions	Number of genes
Chemosensory	3
Immunity	0
Oxidative stress	9
Development	4
P450	3
Circadian Signaling	1
Esterase	2
Serine Protease	1

Table S4. Nucleotide diversity (π) calculated from whole genome sequences in insects. The species were sorted according to π. * denotes π calculated from the sum of π divided by the assembly size in the fall armyworm. ** denotes π calculated from four-fold degenerative sites.

Species	Order	π	Reference
Anopheles moucheti	Diptera	0.0016-0.0631	Fouet et al[90]
Spodoptera frugiperda – sfR	Lepidoptera	0.0443	This study
Spodoptera frugiperda – sfC	Lepidoptera	0.0435	This study
Butterfiles	Lepidoptera	0.0044-0.0428**	Mackintosh et al[91]
Anopheles gambiae	Diptera	0.0301	Corbett-Detig et al[92]
Bombyx mandarina	Lepidoptera	0.0276	Corbett-Detig et al[92]
Spodoptera frugiperda – sfR*	Lepidoptera	0.0239	This study
Spodoptera frugiperda – sfC*	Lepidoptera	0.0235	This study
Heliconius melpomene melpomene	Lepidoptera	0.0185	Corbett-Detig et al[92]
Spodoptera littoralis	Lepidoptera	0.013-0.016	Cheng et al[84]
Helicoverpa	Lepidoptera	0.004-0.010	Anderson et al[93]
Drosophila pseudoobscura	Diptera	0.0087	Corbett-Detig et al[92]
Drosophila melanogaster	Diptera	0.0087	Corbett-Detig et al[92]
Apis mellifera scutellata	Hymenoptera	0.0062	Corbett-Detig et al[92]
