# Supplementary material for: Positive selection alone is sufficient for whole genome differentiation at the early stage of speciation process in the fall armyworm
Source: BMC Evol Biol. 2020 Nov 13;20:152. doi: 10.1186/s12862-020-01715-3 (PMC7663868; doi:10.1186/s12862-020-01715-3)
Supplement: Supplementary file 20 — Additional file 20: Table S2. Genes within genetic outliers of differentiation identified from the mapping against sfR reference genome. [file 12862_2020_1715_MOESM20_ESM.pdf]

Table S2. Genes within genetic outliers of differentiation identified from the mapping against sfR reference genome

gene ID	Gene name
SFRURICE0000000400	POE_DROME Protein purity of essence
SFRURICE0000000401	unknown
SFRURICE0000000460	unknown
SFRURICE0000000510	unknown
SFRURICE0000001614	unknown
SFRURICE0000001897	unknown
SFRURICE0000002022	unknown
SFRURICE0000002025	unknown
SFRURICE0000002026	unknown
SFRURICE0000002045	unknown
SFRURICE0000002049	unknown
SFRURICE0000002058	SNF4Agamma-PA
SFRURICE0000002073	CG41520-PA
SFRURICE0000002095	ACSA_DROME Acetyl-coenzyme A synthetase
SFRURICE0000002102	Su(P)-PA
SFRURICE0000002114	cxe022a
SFRURICE0000002115	CG2930-PD
SFRURICE0000002172	unknown
SFRURICE0000002176	TCF25_MOUSE Transcription factor 25
SFRURICE0000002182	S15A1_RAT Solute carrier family 15 member 1
SFRURICE0000002185	unknown
SFRURICE0000002211	DYC1_CAEEL Dystrophin-like protein 1
SFRURICE0000002214	unknown
SFRURICE0000002240	tpi
SFRURICE0000002241	CG9302-PA
SFRURICE0000002252	CG8314-PA
SFRURICE0000002253	PGRP2_HOLDI Peptidoglycan-recognition protein 2
SFRURICE0000002255	unknown
SFRURICE0000002256	CG8320-PA
SFRURICE0000002271	POLO_DROME Serine/threonine-protein kinase polo
SFRURICE0000002272	SPRR3_RABIT Small proline-rich protein 3
SFRURICE0000002297	unknown
SFRURICE0000002316	CG13917-PD
SFRURICE0000002330	unknown
SFRURICE0000002334	unknown
SFRURICE0000002335	KCNT1_CHICK Potassium channel subfamily T member 1
SFRURICE0000002339	unknown
SFRURICE0000002340	Iris-PA
SFRURICE0000002358	unknown
SFRURICE0000002625	CG31100-PB
SFRURICE0000003127	SP047
SFRURICE0000003128	GAGJ_DROME Nucleic-acid-binding protein from mobile element jockey
SFRURICE0000003833	unknown
SFRURICE0000003911	CG31183-PB
SFRURICE0000004270	RM11_DROME 39S ribosomal protein L11, mitochondrial
SFRURICE0000004307	CG14253-PF
SFRURICE0000004448	p77

SFRURICE0000004470	unknown
SFRURICE0000004500	unknown
SFRURICE0000004929	unknown
SFRURICE0000005012	unknown
SFRURICE0000005014	Jon65Aiii-PA
SFRURICE0000005041	unknown
SFRURICE0000005042	unknown
SFRURICE0000005043	unknown
SFRURICE0000005044	unknown
SFRURICE0000005045	unknown
SFRURICE0000005046	HARB1_RAT Putative nuclease HARBI1
SFRURICE0000005109	unknown
SFRURICE0000005147	unknown
SFRURICE0000005148	ZBED1_HUMAN Zinc finger BED domain-containing protein 1
SFRURICE0000005157	unknown
SFRURICE0000005158	unknown
SFRURICE0000005159	unknown
SFRURICE0000005166	unknown
SFRURICE0000005167	unknown
SFRURICE0000005168	unknown
SFRURICE0000005188	l(1)G0289-PC
SFRURICE0000005225	Jarid2-PD
SFRURICE0000005345	unknown
SFRURICE0000005778	Y3556_DROME Uncharacterized protein CG3556
SFRURICE0000005838	unknown
SFRURICE0000005939	GNAL_DROME Guanine nucleotide-binding protein subunit alpha homolog
SFRURICE0000006018	mRpL50-PA
SFRURICE0000006095	bma-PE
SFRURICE0000006119	PGRP
SFRURICE0000006128	NR2E1_XENLA Nuclear receptor subfamily 2 group E member 1
SFRURICE0000006151	CG42258-PC
SFRURICE0000006157	TSN
SFRURICE0000006184	Trh-PA
SFRURICE0000006204	unknown
SFRURICE0000006205	unknown
SFRURICE0000006245	CG4928-PC
SFRURICE0000006256	HARB1_BOVIN Putative nuclease HARBI1
SFRURICE0000006261	Ddr-PG
SFRURICE0000006262	unknown
SFRURICE0000006263	Rrp6-PC
SFRURICE0000006322	HTP
SFRURICE0000007150	unknown
SFRURICE0000007496	sl-PA
SFRURICE0000007497	babo-PA
SFRURICE0000007498	unknown
SFRURICE0000007533	Fhos-PG
SFRURICE0000007545	unknown
SFRURICE0000007555	unknown
SFRURICE0000008580	unknown

SFRURICE0000008581	unknown
SFRURICE0000008589	unknown
SFRURICE0000008670	unknown
SFRURICE0000008679	CG9503-PA
SFRURICE0000008873	unknown
SFRURICE0000009171	unknown
SFRURICE0000009172	CATA_LACSK Catalase
SFRURICE0000009286	HTP
SFRURICE0000009527	Lmx
SFRURICE0000009545	unknown
SFRURICE0000009564	CG13293-PC
SFRURICE0000009589	unknown
SFRURICE0000009602	unknown
SFRURICE0000009992	unknown
SFRURICE0000010006	p77
SFRURICE0000010007	Esp-PB
SFRURICE0000010268	ATTY_RAT Tyrosine aminotransferase
SFRURICE0000010479	unknown
SFRURICE0000010572	DNJ60_DROME DnaJ-like protein 60
SFRURICE0000010669	unknown
SFRURICE0000010670	unknown
SFRURICE0000010671	unknown
SFRURICE0000010672	unknown
SFRURICE0000011119	CG30100-PB
SFRURICE0000011120	LRC71_HUMAN Leucine-rich repeat-containing protein 71
SFRURICE0000011121	unknown
SFRURICE0000011470	CYP4L9
SFRURICE0000011557	unknown
SFRURICE0000011575	unknown
SFRURICE0000012296	unknown
SFRURICE0000012297	unknown
SFRURICE0000012298	unknown
SFRURICE0000013185	unknown
SFRURICE0000013186	Gyf-PF
SFRURICE0000013195	unknown
SFRURICE0000013242	CYC
SFRURICE0000013269	COPB2_DROME Coatomer subunit beta'
SFRURICE0000013272	NPRL2_DROME GATOR complex protein NPRL2
SFRURICE0000013514	unknown
SFRURICE0000013769	TIGD4_MOUSE Tigger transposable element-derived protein 4
SFRURICE0000014448	CG31974-PC
SFRURICE0000014634	PGBD4_HUMAN PiggyBac transposable element-derived protein 4
SFRURICE0000015028	unknown
SFRURICE0000015077	unknown
SFRURICE0000015341	POL4_DROME Retrovirus-related Pol polyprotein from transposon 412
SFRURICE0000015610	unknown
SFRURICE0000015795	unknown
SFRURICE0000015895	ARP6_CHICK Actin-related protein 6
SFRURICE0000016153	unknown

SFRURICE0000016269	CG2970-PB
SFRURICE0000016270	CC112_MACFA Coiled-coil domain-containing protein 112
SFRURICE0000016671	ATC1_ANOGA Calcium-transporting ATPase sarcoplasmic/endoplasmic reticulum type
SFRURICE0000016672	unknown
SFRURICE0000016673	TANT_DROME Protein tantalus
SFRURICE0000016674	NEUFC_DROPS Neuferricin homolog
SFRURICE0000016675	acyl
SFRURICE0000016676	GGNB2_DROME Gametogenetin-binding protein 2-like
SFRURICE0000016681	AL7A1_CAEEL Putative aldehyde dehydrogenase family 7 member A1 homolog
SFRURICE0000016683	LR74A_HUMAN Leucine-rich repeat-containing protein 74A
SFRURICE0000016967	unknown
SFRURICE0000017102	unknown
SFRURICE0000017103	unknown
SFRURICE0000017177	HTP
SFRURICE0000017334	CG44247-PB
SFRURICE0000017335	DopEcR-PC
SFRURICE0000017346	DYH6_HUMAN Dynein heavy chain 6, axonemal
SFRURICE0000017348	unknown
SFRURICE0000017354	unknown
SFRURICE0000017373	unknown
SFRURICE0000017374	unknown
SFRURICE0000017385	parvin-PB
SFRURICE0000017391	CG12084-PB
SFRURICE0000017392	unknown
SFRURICE0000017393	unknown
SFRURICE0000017394	unknown
SFRURICE0000017396	GR10
SFRURICE0000017400	BAG6A_XENLA Large proline-rich protein bag6-A
SFRURICE0000017583	dpr20-PA
SFRURICE0000017610	unknown
SFRURICE0000018020	unknown
SFRURICE0000018050	unknown
SFRURICE0000018054	unknown
SFRURICE0000018081	unknown
SFRURICE0000018082	unknown
SFRURICE0000018517	GR146
SFRURICE0000018518	GR140
SFRURICE0000018584	mgl-PE
SFRURICE0000018712	PGS2_CHICK Decorin
SFRURICE0000019020	OSGP2_RAT Probable tRNA N6-adenosine threonylcarbamoyltransferase, mitochondrial
SFRURICE0000019161	unknown
SFRURICE0000019168	futsch
SFRURICE0000019239	unknown
SFRURICE0000019320	DSCAM
SFRURICE0000019609	unknown
SFRURICE0000019614	b6-PA
SFRURICE0000019708	unknown
SFRURICE0000019955	ENV_NPVLD Envelope fusion protein
SFRURICE0000020106	unknown

SFRURICE0000020108	unknown
SFRURICE0000020186	unknown
SFRURICE0000020187	RTJK_DROME RNA-directed DNA polymerase from mobile element jockey
SFRURICE0000020194	unknown
SFRURICE0000020195	Y2R2_DROME Putative 115 kDa protein in type-1 retrotransposable element R1DM
SFRURICE0000020196	unknown
SFRURICE0000020197	unknown
SFRURICE0000020217	unknown
SFRURICE0000020218	unknown
SFRURICE0000020266	CG8177-PA
SFRURICE0000020376	unknown
SFRURICE0000020399	spn
SFRURICE0000020400	unknown
SFRURICE0000020401	HTP
SFRURICE0000020488	CG18547-PA
SFRURICE0000020871	HTP
SFRURICE0000020872	RTXE_DROME Probable RNA-directed DNA polymerase from transposon X-element
SFRURICE0000020907	unknown
SFRURICE0000021181	ENV_NPVLD Envelope fusion protein
SFRURICE0000021402	unknown
SFRURICE0000021473	unknown
SFRURICE0000021518	ppk13-PA
SFRURICE0000021798	unknown
SFRURICE0000021799	unknown
SFRURICE0000021942	unknown
SFRURICE0000021943	CG10904-PB
SFRURICE0000022586	CG6178-PA
SFRURICE0000022670	Sbf-PA
SFRURICE0000022671	unknown
SFRURICE0000022713	unknown
SFRURICE0000022714	CYP4AU2
SFRURICE0000022911	ADCY8_HUMAN Adenylate cyclase type 8
SFRURICE0000023067	unknown
SFRURICE0000023093	p77
SFRURICE0000023266	unknown
SFRURICE0000023267	CG5953-PD
SFRURICE0000023268	unknown
SFRURICE0000023269	unknown
SFRURICE0000023270	ARC1_DROME Activity-regulated cytoskeleton associated protein 1
SFRURICE0000023314	unknown
SFRURICE0000023838	unknown
SFRURICE0000023969	unknown
SFRURICE0000023970	unknown
SFRURICE0000023994	unknown
SFRURICE0000023995	unknown
SFRURICE0000023996	unknown
SFRURICE0000023997	unknown
SFRURICE0000023999	unknown
SFRURICE0000024005	unknown

SFRURICE0000024766	Iris-PA
SFRURICE0000025293	GPRK1_DROME G protein-coupled receptor kinase 1
SFRURICE0000025295	PPIL6_HUMAN Peptidyl-prolyl cis-trans isomerase-like 6
SFRURICE0000025296	HEAT6_DANRE HEAT repeat-containing protein 6
SFRURICE0000025880	unknown
SFRURICE0000026019	unknown
SFRURICE0000026534	unknown
SFRURICE0000026715	RTJK_DROFU RNA-directed DNA polymerase from mobile element jockey
SFRURICE0000026944	unknown
SFRURICE0000027555	unknown
SFRURICE0000027556	unknown
SFRURICE0000027889	HTP
SFRURICE0000027962	unknown
SFRURICE0000027963	unknown
SFRURICE0000028011	PI4KIIIalpha-PC
SFRURICE0000028129	unknown
SFRURICE0000028494	cxe001k
SFRURICE0000029158	unknown
SFRURICE0000029226	CYP340AA2P
SFRURICE0000029347	HTP
SFRURICE0000029628	unknown
SFRURICE0000029671	CG9572-PB
SFRURICE0000029672	unknown
SFRURICE0000030208	unknown
SFRURICE0000030209	unknown
SFRURICE0000030239	PB1_MOUSE Protein polybromo-1
SFRURICE0000030298	unknown
SFRURICE0000030473	unknown
SFRURICE0000030476	unknown
SFRURICE0000030477	RTJK_DROME RNA-directed DNA polymerase from mobile element jockey
SFRURICE0000030502	CG9248-PC
SFRURICE0000030959	SYEP_DROME Bifunctional glutamate/proline--tRNA ligase
SFRURICE0000031106	unknown
SFRURICE0000031293	Taz-PD
SFRURICE0000031294	unknown
SFRURICE0000031295	EDC3_DANRE Enhancer of mRNA-decapping protein 3
SFRURICE0000031296	ERMP1_MOUSE Endoplasmic reticulum metallopeptidase 1
SFRURICE0000031297	Bsg-PB
SFRURICE0000031299	Gbeta5-PA
SFRURICE0000031300	PCH2_DANRE Pachytene checkpoint protein 2 homolog
SFRURICE0000031302	CG6479-PA
SFRURICE0000031303	unknown
SFRURICE0000031391	Dop1R1-PC
SFRURICE0000031759	unknown
SFRURICE0000031760	unknown
SFRURICE0000032013	unknown
SFRURICE0000032055	unknown
SFRURICE0000032091	unknown
SFRURICE0000032092	unknown

SFRURICE0000032367	unknown
SFRURICE0000032368	unknown
SFRURICE0000032415	unknown
SFRURICE0000032416	unknown
SFRURICE0000032777	POLR1_ARATH Retrovirus-related Pol polyprotein from transposon RE1
